# Silver Oxide Reduction Chemistry in an Alkane Environment

**DOI:** 10.1021/acsami.5c01780

**Published:** 2025-04-29

**Authors:** Fayez Alfayez, Mikhail Agrachev, Fabian Matter, Sandro Lehner, Arvindh Sekar, Walter Caseri, Rudolf Hufenus, Sabyasachi Gaan, Manfred P. Heuberger

**Affiliations:** † Department of Advanced Fibers, Empa Swiss Federal Laboratories for Materials Science and Technology, St Gallen CH-9014, Switzerland; ‡ Department of Materials, ETH Zürich, Zürich CH-8093, Switzerland; § Department of Chemistry and Applied Biosciences, ETH Zurich, Zürich CH-8093, Switzerland

**Keywords:** silver oxide, hydrocarbons, polymer, alkane, reduction, mechanism

## Abstract

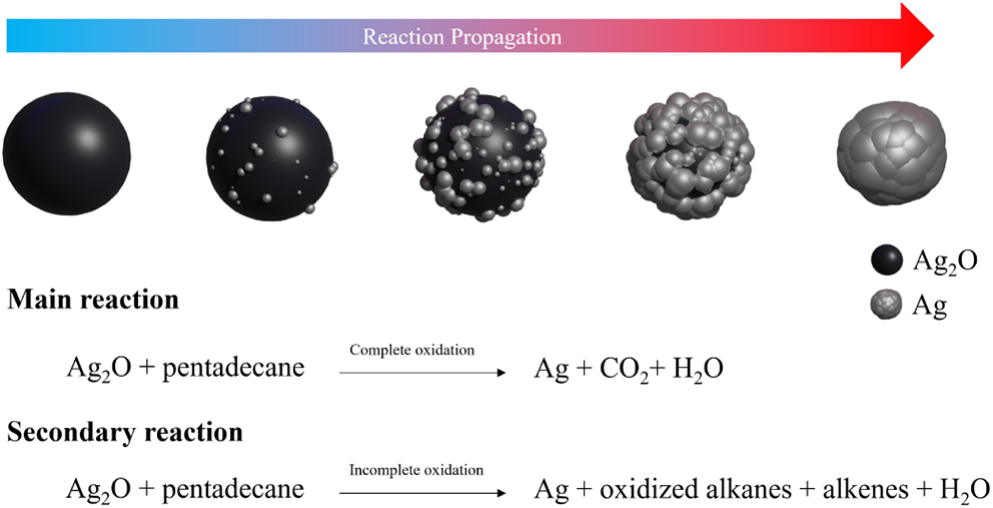

The in situ reduction of silver oxide to metallic silver is of technological relevance for various applications, from conductive welding in electronics to silver (Ag) nanoparticle generation in reactive melt extrusion. This study revisits the redox reaction mechanisms involved in forming metallic silver particles through the reduction of silver(I) oxide (Ag_2_O) in an alkane and polymer melt environment and sheds light on the obtained particulate morphology. Liquid pentadecane was selected as a model alkane, and the observed redox chemistry and particle morphology were compared to the reactive melt extrusion in polyethylene. Unlike the well-studied reduction of metal salts in the presence of oxygen-containing organic materials, the reduction occurring in a pure alkane environment, namely the different particle morphology, is poorly understood. Our findings revealed that the primary byproducts of the reaction between Ag_2_O and pentadecane were CO_2_ and H_2_O, with minor products including alkenes and oxidized alkanes. The reduction process was not linear, with Ag_2_O acting both as a radical initiator and a source of oxygen. Gas chromatography detected CO_2_ formation at a rather low temperature, as low as 70 °C during the reaction between Ag_2_O and pentadecane, indicating a highly oxidative process resembling catalyzed combustion. Analytical techniques, including electron paramagnetic resonance (EPR) spectroscopy, confirmed that radicals were involved in the redox process via ROO• and HOO• radical species typically found in hydrocarbon oxidation under oxygen conditions. We hypothesize that the reaction is predominantly a complete oxidation, with only a small fraction of incomplete oxidation. Our observations also indicated that the metallic Ag formed directly on the surface of Ag_2_O in what appeared to be a solid–solid surface reaction, leading to a final Ag morphology resembling fused particles. While the resulting morphology may seem suboptimal regarding particle dispersion and homogeneity, it still offers a large contact area percolated structure that is advantageous for applications such as electronics welding. We thus conclude that in a pure alkane environment, the redox reactions are confined to the surface of the original particles.

## 1 Introduction

Composites of organic polymers and inorganic metal nanoparticles have been established for various applications, such as thermal management in electronic devices,^1−3^ and antimicrobial protection in medical applications.^4,5^ Traditionally, the fabrication of such nanocomposites includes chemical methods, where metal precursors are reduced in the presence of solvents and reducing agents,^6^ and physical methods like coating and spraying.^7,8^ A prominent example of a chemical method is the polyol process, which utilizes glycols to both reduce and stabilize metal nanoparticles in a solvent medium.^6^ Despite its effectiveness in controlling nanoparticle geometry, this process generates considerable solvent waste. Similarly, the sol–gel technique provides nanoscale control over inorganic structures,^9^ yet both methods are resource-intensive and primarily suited for niche applications.^9,10^ Moreover, a purification step and surface functionalization are generally required to use such metal particles in polymers.

Against this backdrop, the reactive extrusion process presents a promising one-step alternative, which does not require solvents.^11−13^ Under controlled conditions, reactive extrusion is readily applicable for mass production. This process allows chemical reactions, such as the reduction of metal salts, to occur directly inside the extruder, which shapes thermoplastic polymers into useful functional composites. Notably, reactive extrusion has been applied to reduce Ag_2_O in melts of common polymers like polypropylene (PP), polylactic acid (PLA), and polyamide 6 (PA6).^14^ In their investigation, Ag_2_O was chosen as the starting material for nanoparticle generation due to its high Ag content. High loadings of Ag_2_O could be incorporated in the polymer matrix, followed by quick formation of metal particles embedded within the polymer matrix. The resultant composites exhibited excellent Ag ion release properties for antimicrobial usage. The specific mechanisms by which reduction of Ag_2_O to Ag occurs in the melt, particularly in simple polymers lacking functional groups (such as −OH, −C=O, etc.), remain an open question. In contrast, the reduction of Ag salts in the presence of alcohols and aldehydes has been studied.^15^ The reduction is suggested to occur primarily via an ionic pathway,^6,16^ at high pH value, though a study has demonstrated the involvement of a radical pathway,^17^ at neutral pH conditions. Additionally, metal nanoparticles are known to catalyze the oxidation of organic species,^8^ thereby improving the rate of redox reactions. This catalytic behavior is not limited to nanoparticles; metal ions are also used to promote the formation of free radicals.^18^ Regarding the reactivity of alkanes, processes such as combustion,^19^ pyrolysis,^20^ and autoxidation,^21^ are typically explained to involve free radical mechanisms.

A noteworthy study used the naturally occurring oxidative drying process of vegetable oils (unsaturated fatty acid) to reduce AgNO_3_ to Ag nanoparticles.^22^ The reduction process was found to occur via a free radical mechanism, as confirmed by adding either the radical scavenger dimethyl sulfoxide (DMSO) or the radical initiator Fe^2+^. In the presence of DMSO, no reduction occurred even after several months at room temperature. In contrast, when a radical initiator was added, the reduction time decreased 3-fold, from 360 to 120 min.

We previously demonstrated that Ag_2_O can be reduced in commercial-grade polypropylene (PP) during reactive extrusion.^14^ The underlying mechanism of the reduction of Ag_2_O in PP does not appear to involve simple electron pair transfer. The proposed ionic mechanism used for alcohol is based on a reaction where an oxygen atom abstracts a hydrogen atom from the hydroxyl group, however, in our case, alcohols are absent. The basicity of an oxygen atom in Ag_2_O is insufficient to abstract a hydrogen atom from a hydrocarbon; thus, there is a need to investigate the potential intermediates under consideration of free radical formation.

In this study, we aim to investigate the reductions of silver oxide in alkane environments. Instead of PP, we use polyethylene (PE) as a simpler alkane-like polymer. We initially compound PE with Ag_2_O to confirm that PE alone can induce a reduction under extrusion conditions. Differential scanning calorimetry (DSC) and thermogravimetric analysis (TGA) are then used to determine the state of the Ag_2_O. Moreover, pentadecane is used as a model alkane to investigate the reaction intermediates at different temperatures using gas chromatography (GC), Fourier transform infrared (FTIR) spectroscopy, and nuclear magnetic resonance (NMR) spectroscopy. Gas chromatography with a thermal conductivity detector (GC-TCD) is used to quantify the amount of CO_2_, while Karl Fischer titration (KFT) is employed to quantify the amount of H_2_O. Scanning electron microscopy (SEM) coupled with energy-dispersive X-ray spectrometry (EDX) is used to study the structure of the inorganic phase. Electron paramagnetic resonance (EPR) spectroscopy is used to confirm radical formation. Finally, a mechanism for the reduction of Ag_2_O in hydrocarbon is proposed, linking the organic and inorganic phases. This understanding helps bridge the knowledge gap regarding metal oxide and alkane reactions and should also facilitate the development of lead-free solder pastes, where the interfacial fusion of silver particles results in strong, solid connections.

## 2 Materials

Silver(I) oxide was purchased from (abcr) with a purity level of 99%. Prior to use, it was heated at 220 °C in a flask under Ar flow for 5 h to remove any silver carbonate (Ag_2_CO_3_); the temperature was selected based on a thermal study (Figure S1). All manipulations and storage of Ag_2_O were performed in a glovebox. All other chemicals were purchased from Sigma-Aldrich with purity levels as follows: pentadecane (≥99.0%), decanol (≥99.0%), anhydrous ethylene glycol (99.8%), decanoic acid (≥99.5%), and anhydrous methanol (≥99.9%). Bubbling through with Ar and subsequent Schlenk line manipulation were used to remove air from the pentadecane. 3Å molecular sieves were used to dry and store the pentadecane. Polyethylene (PE) powder was purchased from Sigma-Aldrich with an average *M*_w_∼4000. A low molecular weight PE was used to reduce shear-induced chain scission inside the extruder.

## 3 Methods

### 3.1 Physical Analysis in Polymer Melt

Differential scanning calorimetry (DSC) was conducted using a DSC equipment (214 Polyma, Netzsch, Germany) to determine the reduction temperature. The sample size was approximately 5 mg, and the heating rate was set to 10 °C/min. Aluminum crucibles with a perforated lid were used, and the samples were kept under a constant N_2_ flow of 50 mL/min. Similarly, thermogravimetric analysis (TGA) was carried out (TG 209 Libra, Netzsch, Germany) to observe the Ag_2_CO_3_ decomposition temperature and to find the onset and peak thermal reduction temperature of Ag_2_O. The sample weight was kept at 5 mg, and alumina crucibles were used under a N_2_ flow of 50 mL/min. The heating rate was set to 10 °C/min.

PE powder was manually premixed with Ag_2_O powder at a 10 wt % Ag_2_O. Reactive extrusion was carried out using a tabletop compounder (MC 15 HT, Xplore, Netherlands). To minimize the effect of oxygen, a constant flow of Ar gas was fed into the top of the vertical compounder during the compounding process. The compounding temperature was set to 180 °C, and the premix sample was then compounded for 3 and 20 min. The 3 min-interval represents a well-mixed blend with partially converted Ag_2_O, while the 20 min-interval corresponds to the complete reduction of the inorganic phase, accompanied by a noticeable change in color. Next, 5 mg of the collected samples were tested in the DSC to quantify Ag_2_O reduction. The sample heating rate was set to 10 °C/min, and the samples were kept under a N_2_ flow of 50 mL/min.

To examine the morphology of Ag and Ag_2_O particles, scanning electron microscopy with energy dispersive X-ray spectrometry (SEM-EDX) was used (S-4800, Hitachi, Japan). To extract the solid phase, the compounded samples were dissolved in toluene at 80 °C for an hour, then spin-casted at 1000 rpm for 15 min onto a silicon wafer (p-type boron doped). As the liquid was expelled, the wafer was transferred to a vacuum oven set at room temperature, with the pressure maintained at 2 mbar for 1 h. After drying, the sample was mounted onto an SEM stub without the addition of a conductive coating.

### 3.2 Physical Analysis in Model Liquids

To study the temperature dependence of the reduction kinetics, Ag_2_O was added to pentadecane in a 1:1 mol ratio and analyzed using DSC. Samples of 5 mg were analyzed at different heating rates (5, 10, 15, 20 °C/min) from 25 to 230 °C under a N_2_ flow of 50 mL/min (Figure S2). A Kissinger plot was constructed to determine the activation energy of the reaction; moreover, to evaluate the activation energy as a function of conversion, the Ozawa-Flynn-Wall method was applied (Figure S3). The complete conversion time of Ag_2_O to Ag in pentadecane at 180 °C was found to be approximately 30 min. Thus, several experiments were conducted at this temperature. In addition to pentadecane, several other functional alkanes were tested with Ag_2_O in the DSC. The morphology of Ag_2_O and Ag was examined using SEM/EDX. Sample preparation for SEM analysis was performed using a standard spin-cast procedure as previously described.

### 3.3 Chemical Analysis in Model Alkane

Based on an initial assessment of reduction temperatures, systematic reduction of Ag_2_O in pentadecane was performed using a setup as shown in (Figure S4). The setup consisted of a round-bottom flask connected to a cold trap (acetone/ice slurry maintained at −78 °C). A mixture of 25 mL of pentadecane with 20.9 g of Ag_2_O was placed in the flask, all performed inside the glovebox. The setup was evacuated and purged with Ar three times in a row, followed by continuous purging with Ar. A gas bubbler and a valve were used at the gas outlet to prevent backflow.

The flask was heated to 180 °C for 30 min; during this interval, an exothermic reaction was observed, accompanied by the evolution of gases and vapors, as observed from the evolution of the rate of bubbling. The evolved vapors were collected in the cold trap and analyzed with Fourier transform infrared (FTIR) and nuclear magnetic resonance (NMR) spectroscopy. FTIR spectra (650 cm^–1^ to 3600 cm^–1^) of cold trap products were measured in ATR mode (Tensor 27 FTIR, Bruker, USA). NMR spectra were recorded at 400.2 MHz (AV III 400, Bruker, USA). The ^1^H NMR chemical shift (δ) at 7.26 ppm was taken as the chloroform reference solvent.

Gas chromatography–mass spectrometry (GC-MS), performed in both headspace and liquid injection modes, was used to identify the reaction byproducts and intermediates. The gas chromatograph (Trace 1300, Thermo Fisher, USA), equipped with a quadrupole mass spectrometer (ISQ 7000, Thermo Fisher, USA), an agitator, and an autosampler, was used. The vials for headspace analysis were prepared in a glovebox with a 1:1 mol ratio of pentadecane to Ag_2_O, and the vial screw caps had silicon/PTFE septa capable of withstanding high temperatures. An agitator was used to heat and shake the vials at 180 °C for 30 min before injection into the GC column (DB-WAX, Agilent, USA). After the designated time elapsed, the autosampler took 1000 μL of the headspace sample and injected the gas into the GC column. Individual compounds were identified by comparing their mass spectra with those reported in the 2002 NIST MS Library 2.0 using (Chromeleon 7.2, Thermo Fisher, USA) software.

To determine the evolution of gases such as hydrogen, oxygen, carbon monoxide, and CO_2_, a gas chromatograph with a thermal conductivity detector (GC-TCD) was used. Ar gas was bubbled through a flask containing Ag_2_O and pentadecane at (1:10 mol:mol), with the flask placed in a heated oil bath set at 145 °C (setup shown in Section SI and Figure S5). With reference to the previously used 180 °C, a lower temperature of 145 °C was selected to slow the redox reaction and allow for the collection of more data points. Based on observations CO_2_ started forming at 70 °C during heating up to 145 °C, a measurement was specifically taken at 70 °C to investigate this production.

Karl Fischer titration (KFT) was used to determine the amount of moisture produced, using a (906 Titrando, Metrohm, Switzerland) titrator. Prior to testing, pentadecane was stored with molecular sieves, and the reaction vials were prepared in the glovebox. A measured 167 mg of Ag_2_O was exposed to pentadecane at a 1:10 mol ratio in a closed vial, which was then heated in an oven at 180 °C for 30 min. After the reaction, 1 mL of methanol was added to the vial through the septa, and the mixture was vigorously shaken to ensure thorough mixing of H_2_O and methanol. Then, 0.3 mL of the resulting methanol was extracted and transferred into the KF reactor, where the amount of iodine solution consumed in a neutralizing reaction was measured, allowing for the quantification of the moisture present in the sample.

Electron paramagnetic resonance (EPR) spectroscopy experiments were performed using a custom-built setup for in situ analysis at high temperatures and under gas flow. The following settings were used: (microwave frequency = 9.2 GHz, center field = 3172 G, sweep width = 150 G, modulation amplitude = 0.5 G, modulation frequency = 100 kHz, microwave power = 1.983 mW, power attenuation = 20 dB, conversion time = 12.05 ms, time constant = 10.24 ms). A quartz capillary (inner diameter = 0.8 mm) was placed inside an EPR quartz tube (Wilmad; inner diameter = 2.8 mm), which was loaded with the sample (pentadecane or Ag_2_O/pentadecane suspension). The EPR tube was housed at the center of a homemade water-cooled high-temperature resonator,^23^ which was installed into a continuous wave EPR spectrometer (Bruker EMX) operating at X-band frequencies. N_2_ gas was directed through the central capillary and bubbled through the suspension during the measurement. In situ experiments were carried out while continuously recording EPR spectra using a 2D acquisition mode enabling a time-resolved monitoring of the process. All of the spectra were simulated using the EasySpin package running in MATLAB.^24^

## 4 Results and Discussion

### 4.1 Physical Analysis in Polymer Melts

**Figure 1:**
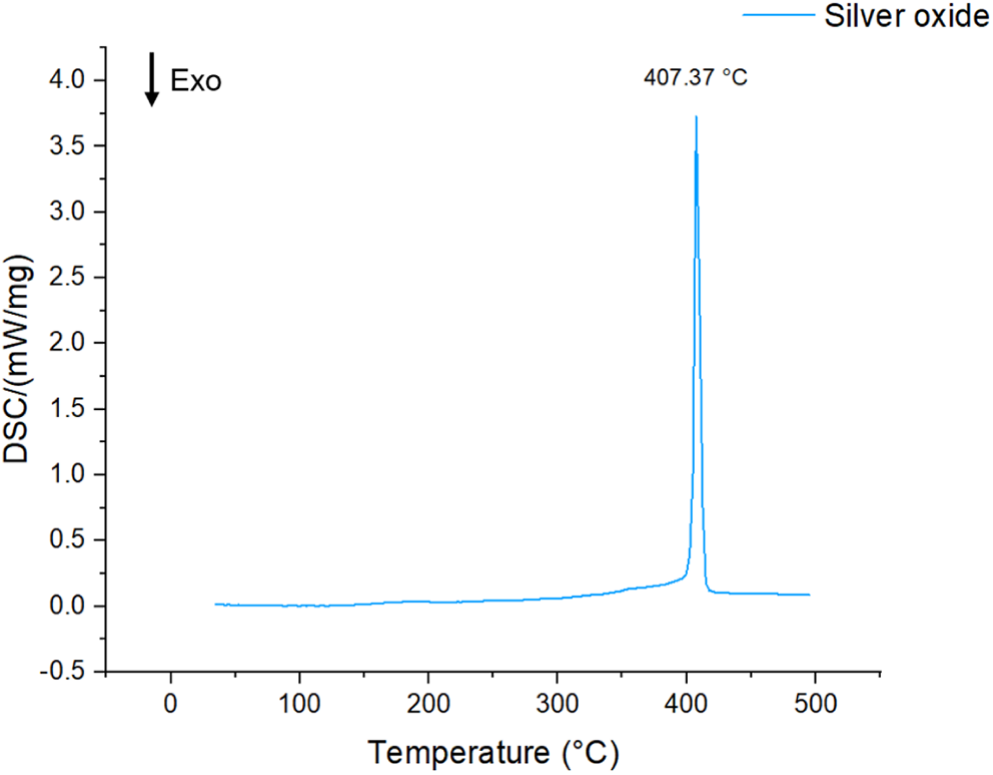
DSC of silver oxide at a 10 °C/min heating rate under N_2_ flow. An endothermal peak, attributed to the decomposition of Ag_2_O, appears at 407 °C.

The Ag_2_O underwent an endothermic reduction to metallic Ag under N_2_ at temperatures of 407 °C (Figure 1) and an onset temperature of 400 °C (Figure S1), which is in line with thermal reduction temperatures reported by others,^25−27^ though some variation could arise due to preparation procedure.^28^

With reference to the application case of reactive extrusion, we also investigated the reduction behavior of Ag_2_O within a molten PE matrix via the reactive extrusion process. In our previous study, we examined the reduction of Ag_2_O in commercial-grade PP, which contained primary and secondary antioxidants and other processing additives.^14^ We note that the tertiary carbon atom in polypropylene is particularly susceptible to oxidative degradation,^29^ which could facilitate the reduction of Ag_2_O and oxygen capture, thereby adding to the reduction pathways. In the current work, low molecular weight virgin PE was chosen for the melt-extrusion process to isolate redox chemical pathways and simultaneously prevent high shear effects (shear-induced chain scission).

**Figure 2:**
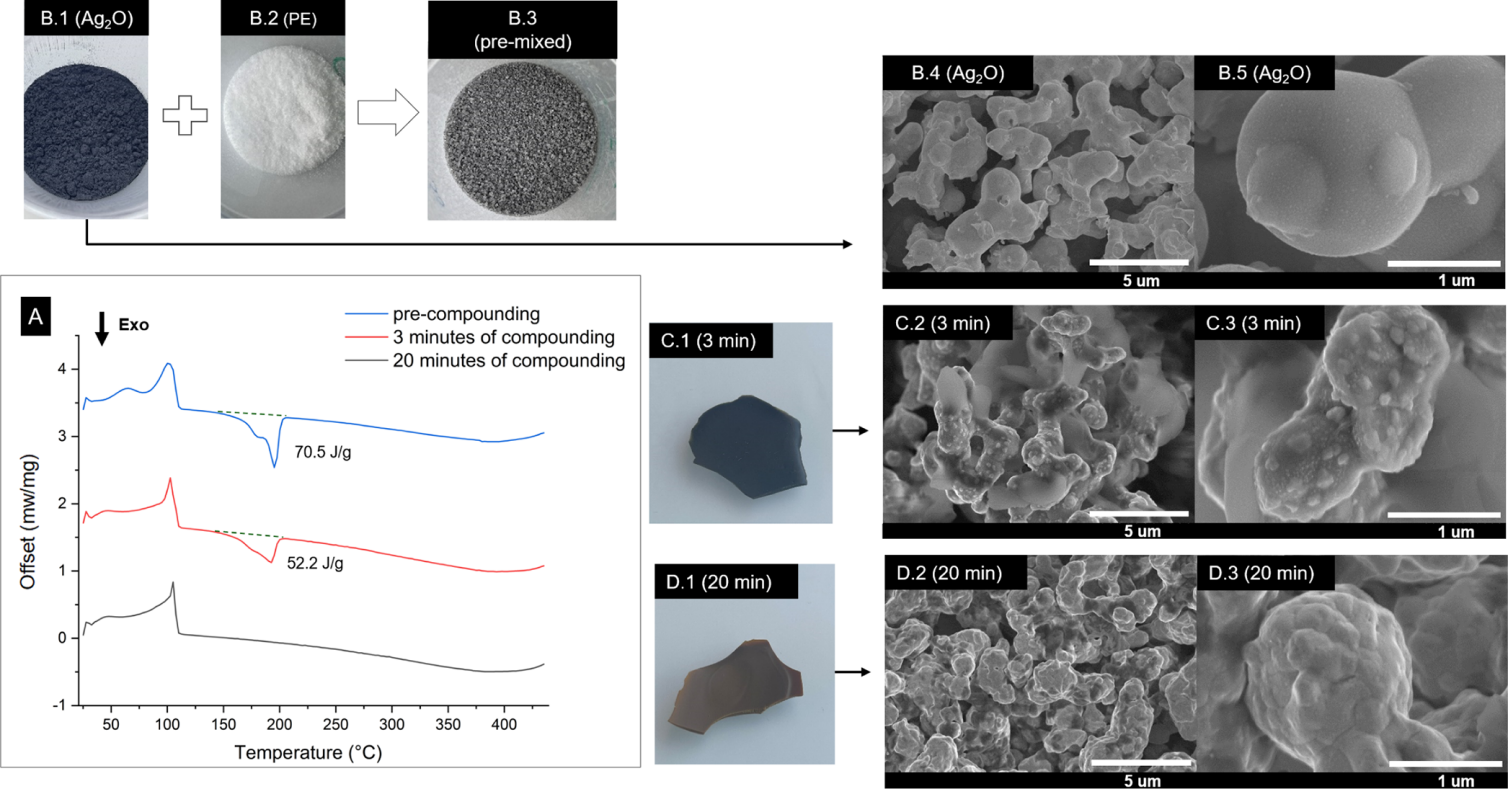
(A) DSC thermograms of three different samples: premixed or compounded in a microextruder for 3 or 20 min. The heating rate used was set to 10 °C/min under N_2_ flow. The premixed powder consists of Ag_2_O (B.1) and polyethylene powder (B.2), forming the mixture seen in (B.3). Figures B.4 and B.5 show SEM images of (B.1). The 3 min compounding extrudate (C.1) contained Ag/Ag_2_O shown in (C.2). The 20 min compounding extrudate (D.1) contained fully reduced Ag shown in (D.2). Higher magnifications SEM images are shown in (B.5, C.3, D.3), corresponding to (B.4, C.2, D.2) respectively. Ag nucleation is observed on Ag_2_O surfaces (C.3).

Dry mixing of Ag_2_O/PE (10 wt % Ag_2_O loading, Figure 2 B.3, labeled as premixed) led to nonuniform gray powder. This sample then was further melt-extruded (compounded) at a temperature of 180 °C for a duration of either 3 or 20 min. The 3 min duration was observed to be the minimum time required to homogenize the Ag_2_O loaded melt, while a 20 min duration in the extruder already showed complete conversion of Ag_2_O to Ag. After 3 min of compounding, the samples turned dark green (Figure 2C.1), and with 20 min of compounding, the samples appeared dark yellow (Figure 2D.1).

To determine the degree of reduction of Ag_2_O to Ag, DSC was performed on the Ag_2_O/PE mixtures namely the premixed, 3 min duration, and 20 min duration. The Ag_2_O/PE thermograms revealed an exothermic peak (Figure 2A) attributed to the heat released during the DSC-induced reduction of Ag_2_O. Notably, while the reduction of Ag_2_O in an inert atmosphere is endothermic, its reduction in PE was exothermic, suggesting a separate reduction pathway. Conversely, the absence of an exothermic peak in DSC indicated that all the Ag_2_O has already been reduced to metallic Ag (Figure 2A). An endothermic peak at 105 °C was observed for all samples, corresponding to the polymer melting temperature. The premixed sample displayed an exothermic peak around 194 °C with an area of 70.5 J/g, quantifying the maximal amount of Ag_2_O that can be reduced during the DSC experiment.

The 3 min compounded sample also exhibited an exothermic peak around 194 °C, but with a lower enthalpy change of 52.2 J/g, suggesting that only a partial reduction occurred. After 20 min of compounding, the exothermic peak around 194 °C was not observed, indicating that the entire Ag_2_O had been converted to metallic Ag; this concurred with a color change to yellow (Figure 2D.1). Detailed morphological investigations of these three samples revealed that metallic Ag predominantly proliferated on the surface of Ag_2_O particles (Figure 2C.3), suggesting a solid-state conversion mechanism where Ag diffusion is mainly limited to the particle surface. As the compounding time increased, initially smooth particle surfaces with rounded edges were transformed into more complex particle shapes, eventually resembling fused agglomerates of smaller particles.

The transformation of Ag_2_O to Ag occurred around 194 °C in polyethylene (PE) melt, which is significantly lower than the 407 °C observed in N_2_. The thermal reduction of Ag_2_O under a nitrogen atmosphere is an endothermic process and energy is required to break the silver–oxygen bonds. However, in a PE melt the process was exothermic due to additional chemical reactions, such as ones leading to high-binding energy products, H_2_O or CO_2_. This highlights the key fact that the polymer melt provided reaction energy and new pathways.

To our knowledge, no previous work has scrutinized the mechanism behind this phenomenon, although some reports have also observed a lowered reduction temperature for different anions, such as AgNO_3_, in polymers with functional groups like poly(vinyl alcohol) or poly(lactic acid).^30−33^ Polymer extrusion introduces several additional variables that could influence the reduction of Ag_2_O to elemental Ag, including shear forces, which are known to increase reaction rates and create localized regions with higher temperatures.^34,35^ Even though we tried to limit the presence of oxygen, small amounts of oxygen are expected to find their way into the extruder and affect the reduction process.

To reduce viscosity and extend the fluid temperature range, we use pentadecane, a low-molecular-weight model hydrocarbon, for systematic mechanistic investigations. Another obvious advantage is that the characterization of reaction intermediates and decomposition products is more straightforward.

### 4.2 Physical Analysis in Model Liquids

**Figure 3:**
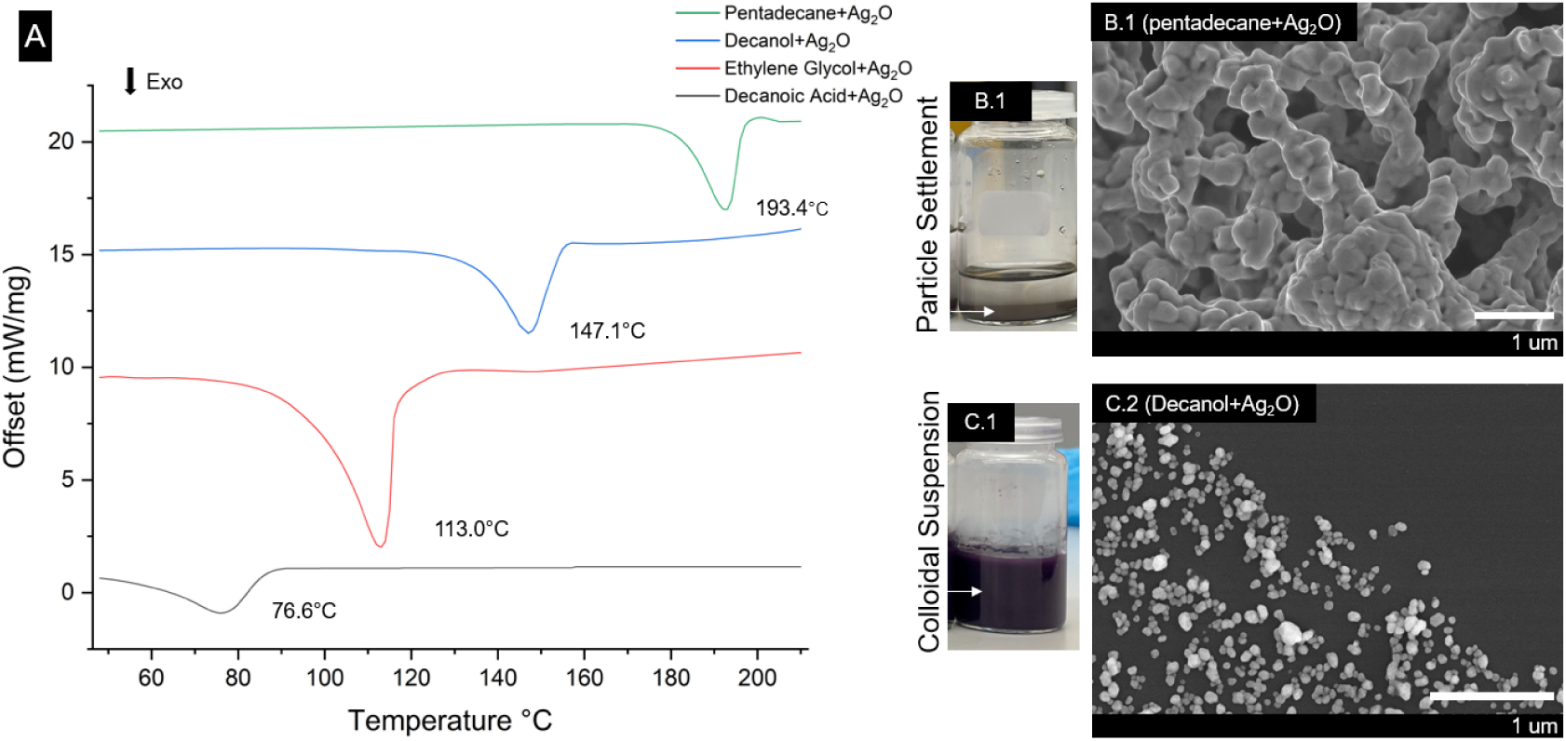
(A) DSC thermogram of Ag_2_O reduction in different organic liquids, showing the exothermic peaks (i.e., down) for the reduction of Ag_2_O with pentadecane, 1-decanol, ethylene glycol, and decanoic acid. The reduction temperatures vary clearly with the functional groups; while pentadecane (pure alkane) exhibits the highest transition temperature, more functional alkanes trigger the transition at even lower temperatures. (B.1) Observed sedimentation of Ag particles and agglomerates. (B.2) Morphology of Ag reduced in pentadecane. (C.1) Colloidal suspension of Ag in 1-decanol, and (C.2) morphology of Ag particles reduced in 1-decanol, showing well-dispersed particles (∼80 nm) with some agglomeration rising from sample preparation for SEM.

**Figure 4:**
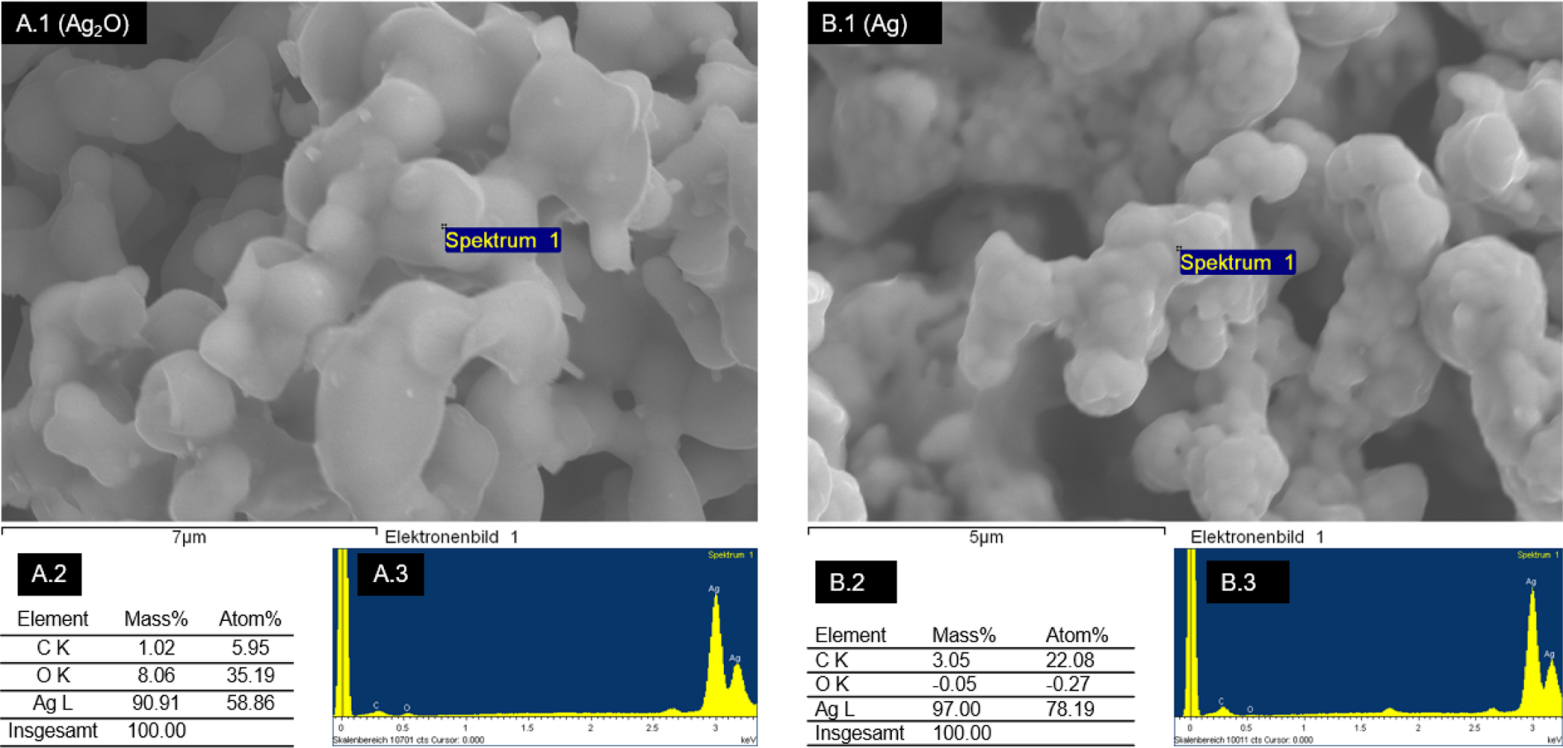
Silver(I) oxide before (A.1) and after (B.1) reduction to metallic Ag in pentadecane, at 180 °C for 30 min under Ar. (A.1) depicts a spot on silver(I) oxide that concurs with the EDX analysis, displaying the elemental distribution Table (A.2) and the characteristic EDX energy spectrum (A.3). On the right, (B.1) depicts the spot on the reduced silver that was selected for EDX, with (B.2) and (B.3) displaying the elemental distribution and energy spectrum, respectively.

The reduction of Ag_2_O in pentadecane and other organic liquids was studied using DSC (Figure 3 A.1). Like the reduction observed in PE melt, pentadecane facilitated the reduction of Ag_2_O at a reduction temperature of 193 °C. Beyond the DSC measurements, the reaction was also conducted in a glassware setup, and the conversion of Ag_2_O to Ag was confirmed by analyzing the residual inorganic phase using EDX, as depicted in Figure 4.

The similarity of the transition temperatures between PE and pentadecane supported the idea that pentadecane indeed served as a model system for olefin polymer melts. The produced Ag particles sedimented and were randomly oriented (Figure 3B.1,2). The morphology resembled agglomerates of fused particles, but it is due to a solid-state reaction involving surface-confined diffusion that occurs significantly below the melting temperature of Ag (962 °C), see Supplementary Video 1 as an example of how this could occur.

Ag_2_O is commonly employed in electronic packaging pastes to generate Ag, which facilitates the formation of strong, conductive bonds at elevated temperatures and pressures (above 200 °C and 5 MPa), depending on the substrate material.^3,36^ This conductive bonding process utilizes reducing solvents such as diethylene glycol (DEG) or polyethylene glycol (PEG), which can control the formation and electrical quality of the bond. In such an application, the goal is to achieve a dense, continuous, void-free Ag layer that forms a reliable mechanical and electrical contact between the substrates.

Reduction in pentadecane led to the formation of large and fused particles which could be useful for electrical welding application. The observed surface diffusion on Ag particles enhances the welding and fusion effect. We bear in mind that the long-term storage stability of alcohol/Ag_2_O pastes is limited, as reduction is expected to occur near room temperature. The slightly higher transition temperature found with alkanes, in combination with the surface-confined transport, offers pathways to formulations of more stable pastes, where initiation is possible with a moderate temperature increase.

The use of alcohols or acids as reducing agents is also established in processes such as the polyol process^8^ and sol–gel processes,^37^ where ethanol, ethylene glycol, or citric acid act as reducing agents and nanoparticle stabilizers. Carbonyl groups (C=O) are also known to reduce metal salts, as in the Tollens’ test. To further assess the role of chemical functional groups, we compared the reduction temperatures of 1-decanoic acid, ethylene glycol, 1-decanol, and pentadecane and observed a clear trend for the peak reduction temperatures at 77 °C, 113 °C, 147 °C, and 193 °C, respectively (Figure 3A.1). The compounds with carboxylic acid or alcohol groups have oxygen atoms that can participate in redox reactions more readily than the hydrogen and carbon atoms in alkanes. This increased reactivity can be attributed to the energetically favored hydrogen abstraction from these functional groups compared to alkanes.

These results thus indicated that the presence of chemical functional groups in organic molecules can further lower the reduction temperature of Ag_2_O. We note that the outcome of the particle morphology is decisively affected by such differences in a chemical environment, as shown in Figure 3B.2,C.2. Namely, the reduction in 1-decanol produced well-dispersed nano-Ag colloids, see Figure 3C.1, suggesting a different transport mechanism and nucleation of the silver from its oxide to the reduced form; this is unlike the case observed in pure alkane environment (i.e., pentadecane) where a particle surface confined reaction was predominantly observed (Figure 3 B.1,C.1). The occurrence of a mobile form of silver in the presence of chemical functional groups is remarkable and relevant for applications that can profit from nanoparticulate dispersions of metallic silver (e.g., antibacterial).

### 4.3 Chemical Analysis in Model Alkane

**Figure 5:**
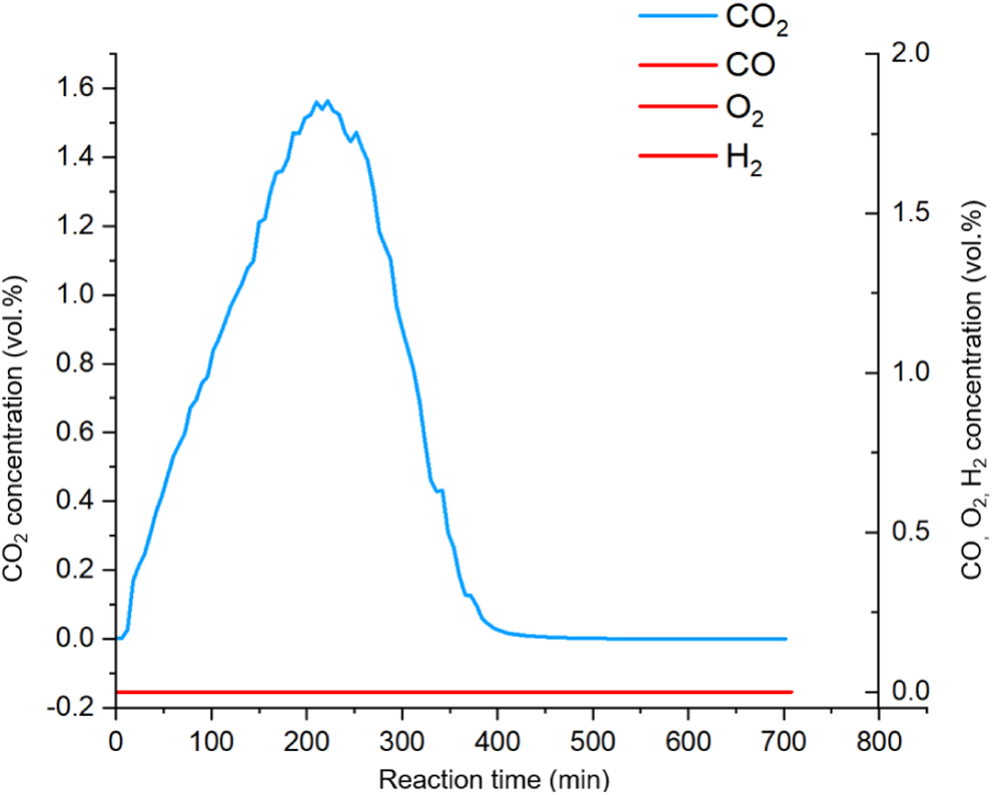
Concentration of the released gases vs time, conducted in a glassware setup connected with GC-TCD, sampling the reaction mixture every 2 min under a continuous flow of Ar (20 mL/min).

To understand the reaction mechanism, we analyzed the gas phase released during the reduction process of Ag_2_O in pentadecane (1:10 mol:mol) by GC-TCD. The recorded data excludes the production of CO, H_2_, or O_2_ (Figure 5); however, significant quantities of CO_2_ and H_2_O were detected (Section SI and Figure S6). The CO_2_ production reached a maximum after 200 min and ended after ca. 400 min (Figure 5). The final decay is possibly due to the decreasing availability of Ag_2_O and the growth of the Ag particle on the surface of Ag_2_O as the reaction proceeds, indicating that the oxygen in the CO_2_ must originate from Ag_2_O. By quantification of the detected CO_2_ (Figure 5) in comparison to the original amount of Ag_2_O, we determined that approximately 61% of the oxygen from Ag_2_O was converted to CO_2_ in a full conversion. The remaining 39% of the oxygen was accounted for by the quantity of H_2_O, measured using the Karl Fischer titration method (see Table 1). As per the GC-TCD results, the amount of moisture should have been 39% however KFT measured 41%. This difference might be due to KFT’s inherent measurement error. It is interesting to note that the H/C elemental ratio is 2.14 in pure pentadecane, while the measured H/C ratio in the gas phase products is 2.56, which suggests that alkane deprotonation may be an important reaction pathway.

**Table 1:** Distribution of the Oxygen of Ag_2_O in CO_2_ and H_2_O, Using GC-TCD and KFT

Product	Amount of Ag_2_O consumed	Method
CO_2_	60.72%	GC-TCD
H_2_O	41.3%	KFT

**Figure 6:**
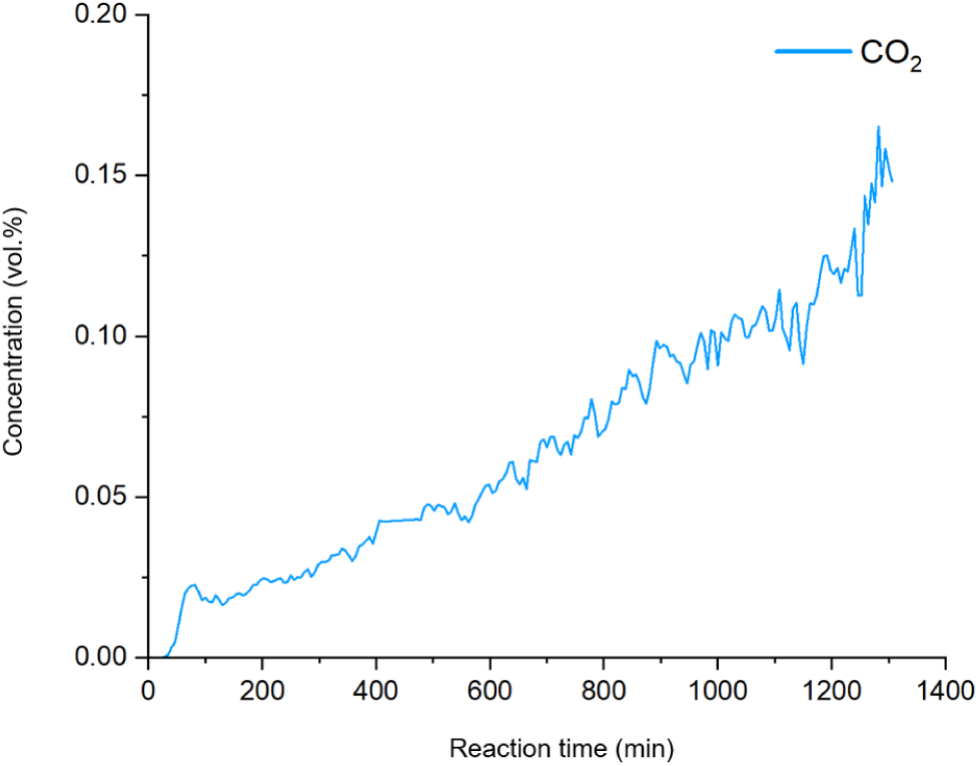
Gas chromatography shows the continuously accelerated CO2 production at 70 °C under a continuous flow of Ar (20 mL/min).

Interestingly, during the heating of the sample containing Ag_2_O and pentadecane to 145 °C, CO_2_ production started at a temperature as low as 70 °C. We thus conducted a comparative study experiment at 70 °C (Figure 6), showing that after an initial burst around 84 min, the CO_2_ production rate (slope in Figure 6) continued to increase even though the temperature was kept constant. This redox reaction occurs at such moderate temperatures only in the presence of Ag_2_O, likely promoted by an autocatalytic effect, possibly relating to an increasing amount of metallic Ag. To exclude that the CO_2_ production at this low temperature could arise simply by thermal decomposition of pentadecane alone, we conducted GC-MS headspace analysis of pentadecane in air, heating the sample to 70 °C for 120 min. As one might expect, no CO_2_ was detected (Section SI and Figure S7), indicating that CO_2_ is only released in the presence of Ag_2_O.

**Figure 7:**
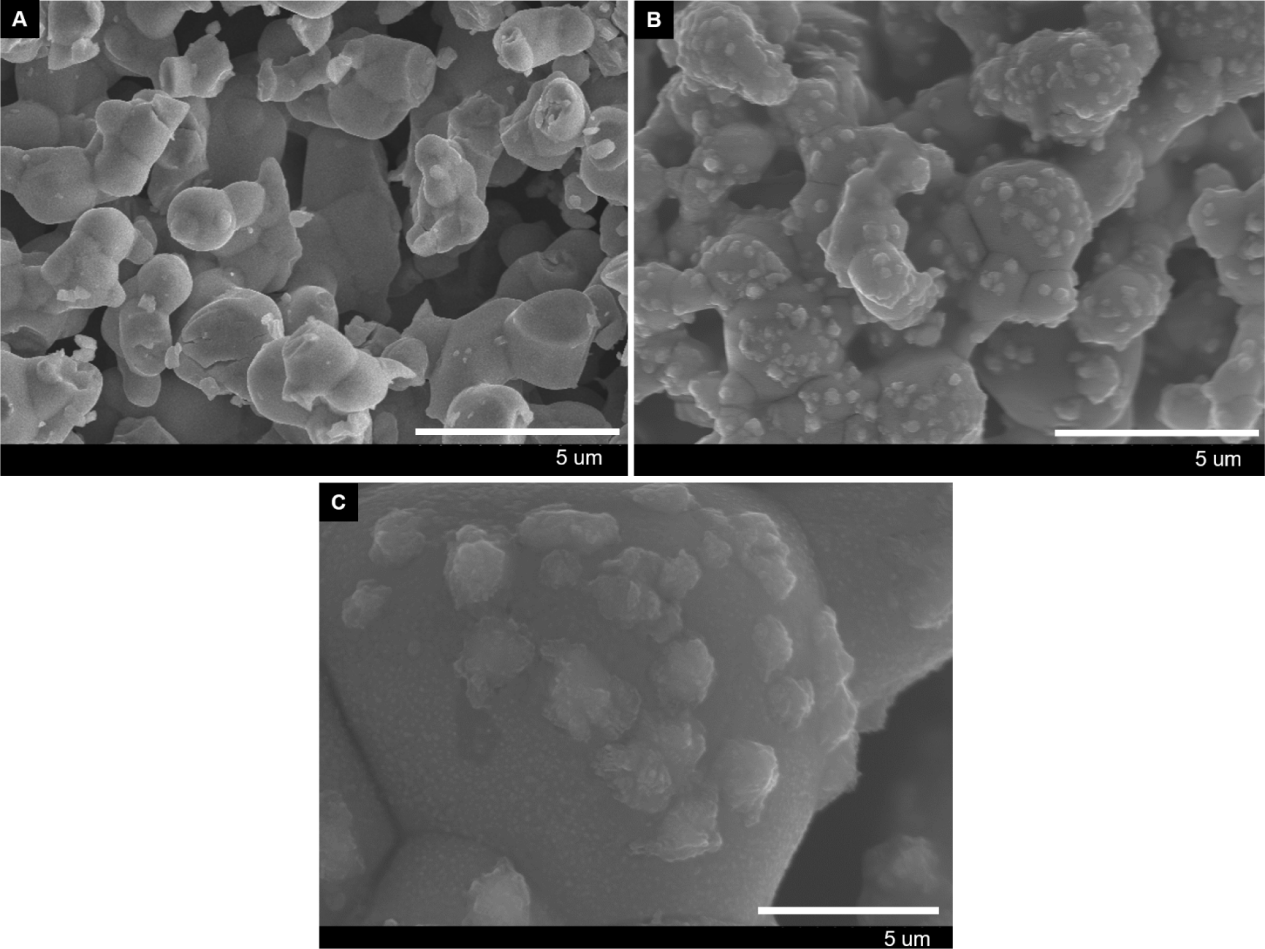
(A) Virgin Ag_2_O and (B) Ag_2_O exposed to pentadecane at 70 °C. (C) Magnified image of (B).

As CO_2_ was produced at the near-ambient temperature of 70 °C during the reaction of Ag_2_O with pentadecane, we revisited the Ag_2_O morphology and compared it to the original Ag_2_O (see Figure 7). Indeed, the morphology of the Ag_2_O showed changes, exhibiting protrusions resembling fused particles appearing at the surface, suggesting again a surface-confined solid-state conversion reaction. This newly formed metallic Ag might play a catalytic role in the accelerated oxidation of the alkane. The earlier observation of a parabolic CO_2_ production rate could be linked to these particle formations.^38^

**Figure 8:**
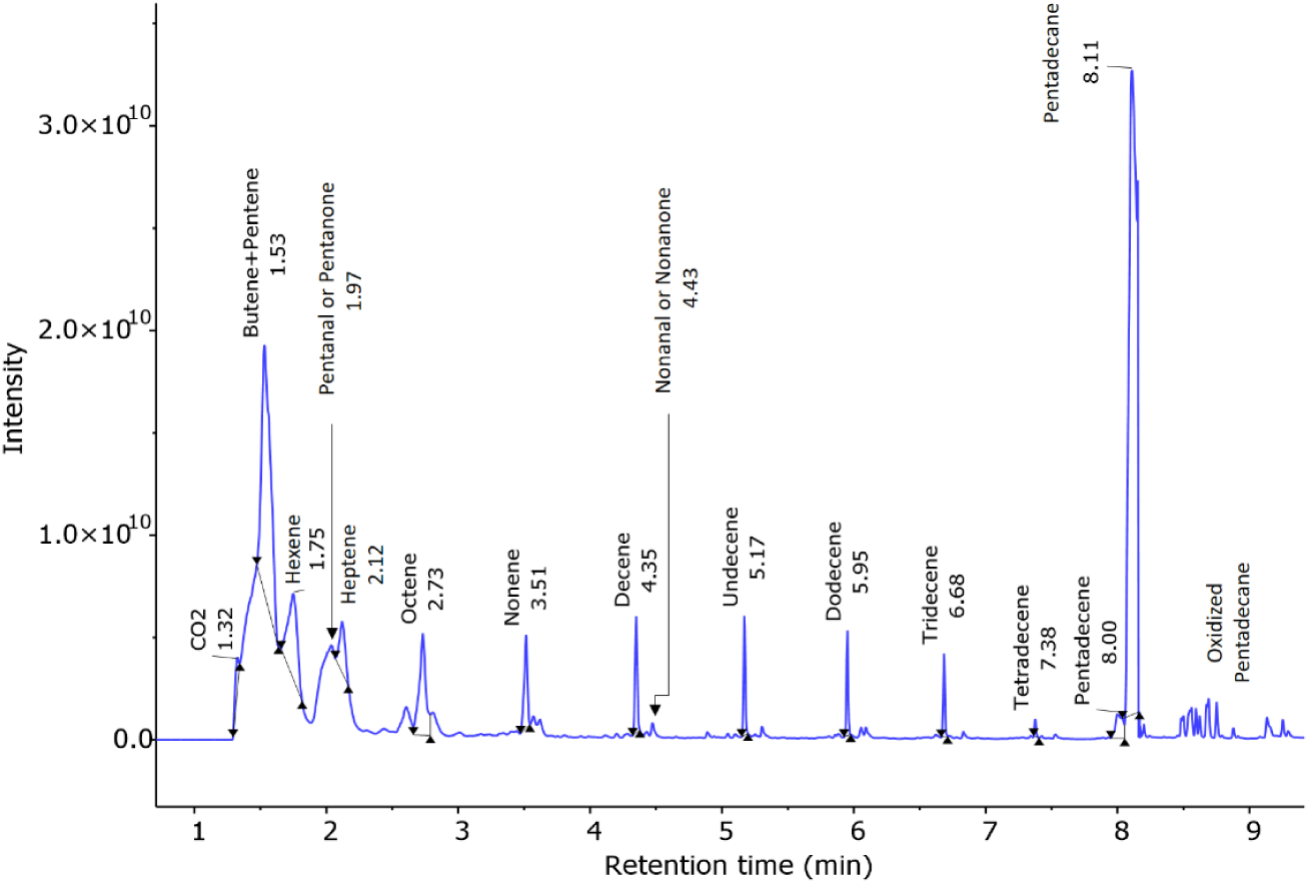
GC-MS full spectrum highlighting the main peaks and their retention time (min).

Moreover, the liquid adducts of the reaction of Ag_2_O and pentadecane at high temperature did not yield any byproducts, as observed in the GC-MS analysis (Section SI and Figure S8). Thus, a headspace gas/vapor analysis was conducted on a mixture of Ag_2_O and pentadecane at 180 °C for 30 min, at a 1:1 ratio (see Figure 8), revealing alkenes of varying lengths, including pentadecene. The spectrum displayed well separated signals with a minor peak surrounding the main peaks.

The first molecular ion of 44 *m*/*z* at a retention time of 1.32 min was attributed to CO_2_. The adjacent peaks occurring at 1.53 min showed molecular ions of 56 and 70 *m*/*z*, corresponding to butene and pentene respectively. Due to their small size, these compounds coeluted, and it was not possible to further determine the position of the unsaturated bond. The next peak at 1.75 min showed a molecular ion of 84 *m*/*z*, indicating hexene. To determine the position of the double bond, we analyzed its fragmentation pattern and compared it with mass spectrum of various hexene isomers. The fragments for 1-hexene (84, 69, 56, 41 *m*/*z*) best matched our results (Section SI and Figure S9), which confirmed a terminal position of the double bond. The peak at 2.12 min with a molecular ion of 98 *m*/*z* indicated heptene. Table 2 summarizes the main peaks.

**Table 2:** Main Compounds Found with Their Respective Retention Times and Molecular Ions

Retention time (minutes)	molecular ion (*m*/*z*)	Compound
1.32	44	CO_2_
1.53	56, 70	butene, pentene
1.75	84	hexene
2.12	98	heptene
2.73	112	octene
3.51	126	nonene
4.35	140	decene
5.17	154	undecene
5.95	168	dodecene
6.68	182	tridecene
7.38	196	tetradecene
8.00	210	pentadecene
8.11	212	pentadecane

Several peaks followed the main pentadecane peak (at retention time 8.11), corresponding to isomers of pentadecanone, with a molecular ion at 226 *m*/*z*, and other forms of oxidized pentadecane. These signals were poorly resolved due to the column’s limited separation of polar compounds. Additionally, small peaks next to each alkene peak indicated oxidized shorter alkanes. For instance, before detection of the signal corresponding to heptene, a broad signal at 1.97 min with a molecular ion of 86 *m*/*z* suggested the presence of pentanal or pentanone. Another example was the peak after decene at 4.43 min, showing a fragment with a molecular ion of 142 *m*/*z*, indicating nonanal or nonanone (Figure 8).

We suspected that minor peaks correspond to oxidized forms of alkanes. Long chain carbonyls, which often undergo McLafferty rearrangement rather than alpha cleavage in mass spectrometry, were also observed. We note that the poor separation of the column for polar compounds and their low concentration limited our investigation at this point. However, at silver oxide to pentadecane ratio of 1:1 we detected less than 0.5% of oxide species and this amount seems to scale with the total amount of oxygen from silver oxide.

Overall, GC-MS data showed that pentadecane decomposed into a cascade of alkenes, oxidized alkanes and CO_2_. The position of the double bond in hexene, heptene, octene, nonene, decene, undecene, and dodecene appeared to be at the chain end, as confirmed by comparing the mass fragments to several isomers from the NIST library. Another study reported alkene formation from the thermal decomposition of hexadecane, showing very similar GC-MS spectra.^20^ This reaction is termed pyrolysis, if it occurs without a source of oxygen. However, the (uncatalyzed) pyrolysis of hexadecane required higher temperatures and prolonged durations (e.g., 330 °C for more than 25 h). At high temperatures, the C–C bonds in alkanes break homolytically, forming alkyl radicals, which then undergo β-scission, generating alkenes along with smaller radicals.

This process can be catalyzed by metal ions, as it follows the free radical mechanism.^20,21^ However, in our investigations into the reaction of Ag_2_O with pentadecane, we observed the formation of pentadecene, an alkene from the same parent molecule. This contrasts with the typical β-scission mechanism, which produces a smaller alkene rather than an alkene of the same parent module, indicating that the process is not pyrolysis. The GC-MS data thus confirms the hypothesis that the reaction chiefly involved deprotonation rather than a C–C session. This is remarkable in view of the higher energy requirement (C–H ∼ 422 kJ/mol, C–C ∼ 370 kJ/mol). Besides the possibility for (auto) catalytic reactions, it has been reported that alkyl radicals can react with oxygen to produce peroxyl radicals, which undergo internal hydrogen abstraction to yield an alkene and hydroperoxyl at high temperatures,^39,40^ where this pathway appeared more probable than the β-scission mechanism. Free radical reactions and direct deprotonation could thus both be mediated by (auto) catalytic reactions at lower temperatures, as observed here.

**Figure 9:**
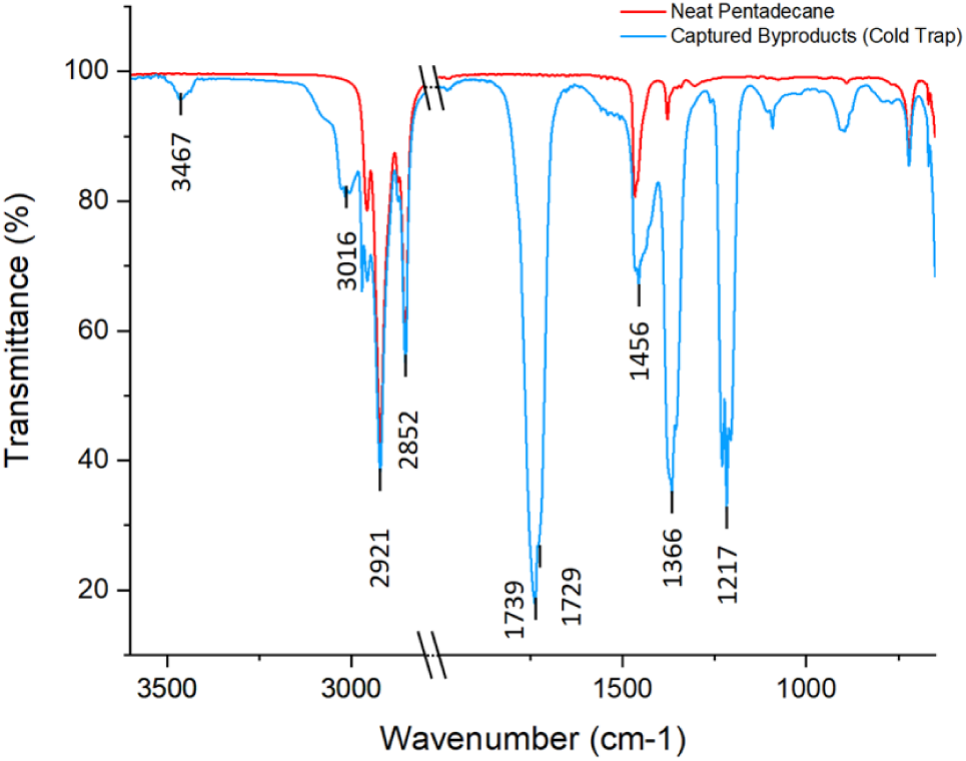
FTIR spectra of the products in the cold trap (blue curve) and pentadecane which was heated to 180 °C for 30 min under Ar atmosphere (blue curve).

To examine the presence of oxidized alkanes, we captured the byproducts using a cold trap. We analyzed them with FTIR and NMR spectroscopy, following the setup shown in Section SI and Figure S5. FTIR spectra were recorded for a cold trap sample and neat pentadecane, see Figure 9. The FTIR spectrum of the products in the cold trap showed a broad peak at 3460 cm^–1^, indicative of O–H bond stretching vibrations, suggesting the presence of alcohol groups. Additionally, a very broad shoulder overlapped by a sharp peak at 3016 cm^–1^ was observed. The broad shoulder was attributed to O–H bond stretching vibrations from carboxylic acids, while the sharp peak at 3016 cm^–1^ corresponded to =C–H stretching vibrations from alkenes, again confirming the prominent presence of alkenes.

Further significant peaks at 2921 cm^–1^ and 2852 cm^–1^ correspond to C–H stretching vibrations typical for hydrocarbons. Major peaks at 1739 cm^–1^ and 1729 cm^–1^ were attributed to C=O stretching vibrations from carboxylic acids and aldehydes, respectively. Although ketones typically exhibit a peak near 1715 cm^–1^, this signal could be overlapped by aldehyde signals. The expected C=C stretching vibration peak in the 1680–1640 cm^–1^ region was not observed, possibly due to overlap with the prominent carbonyl peak. A peak at around 1456 cm^–1^, related to C–H bending vibrations, was observed in both samples, but it was broader and more intense in the cold trap sample, likely due to the presence of O–H bending vibrations in the 1440–1395 cm^– 1^ region. Peaks at 1366 cm^–1^ and 1217 cm^–1^ were associated with methyl group rocking and C–O stretching vibrations from alcohols and carboxylic acids, respectively.

**Figure 10:**
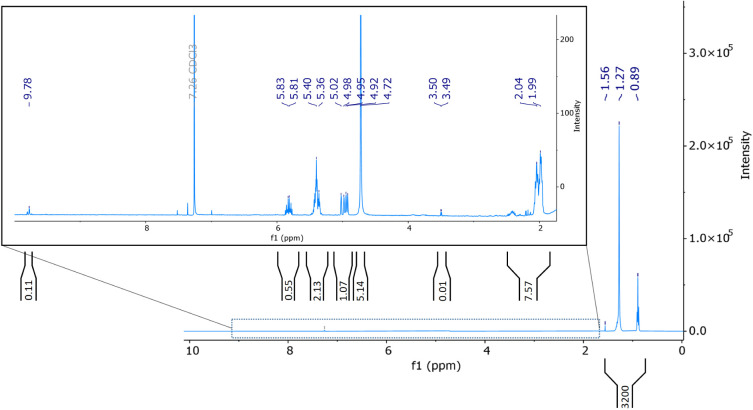
^1^H NMR spectrum (in CDCl_3_) of the cold trap.

The ^1^H NMR spectrum of the cold trap adsorbate showed several peaks, as seen in Figure 10. NMR peaks were typically measured in arbitrary units, with integrated intensities proportional to the number of proton nuclei. The peaks observed at 0.89, 1.27, and 1.56 ppm corresponded to the main proton signals from pentadecane, specifically CH_3_ (methyl) and CH_2_ (methylene) groups. The peak at 1.56 ppm is due to water in CDCl_3_. The area under the curve for the main compound was 3200.

Several peaks appeared in the 1.99 to 2.41 ppm range, which typically belongs to protons of α carbon–hydrogen atoms adjacent to a ketone. Small peaks in this region could also be attributed to hydrogen atoms in beta carbon atoms of aldehydes or carboxylic acids, or gamma protons of alkenes. A doublet was observed at 3.50 and 3.49 ppm with a relative area of 0.01, likely arising from alpha protons of alcohol groups. Additionally, a sharp singlet at 4.72 ppm, with a relative area of 5.14, was attributed to an alcohol proton.

Moreover, several peaks were observed in the 4.9 to 5.9 ppm range, a region characteristic of alkenes. A–CH=CH_2_ unit is represented by two doublets observed at 4.95 and 4.99 ppm with a coupling constant of 12 Hz (*cis*-coupling of a terminal H atom), and at 4.98 and 5.02 ppm with a coupling constant of 16 Hz (*trans*-coupling of a terminal H atom), and a multiplet at 5.82 ppm. Multiplets were observed around 5.38 ppm, belonging to internal alkene groups. The integrated areas imply a ratio between internal and terminal alkene units close to 2. Moreover, a signal at 9.78 ppm and the accompanying smaller signals correspond to protons of α carbon atoms of aldehydes, with a relative area of 0.11. The CHCl_3_ peak in the CDCl_3_ solvent emerges at 7.26 ppm, which served as a reference.

**Scheme 1:**
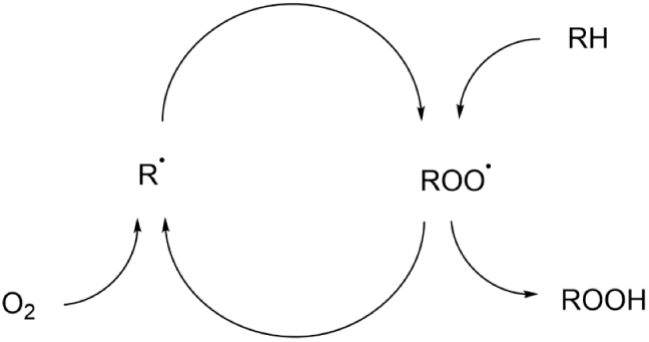
Schematic of Hydrocarbon Autoxidation Steps of Polymers after Radical Formation, Simplified, Adopted From ref (42).

In summary, FTIR and NMR data confirmed the presence of oxidized alkanes, including ketones, aldehydes, carboxylic acids, alcohols, and alkenes, as minor byproducts of the reduction process. For comparison, the known hydrocarbon autoxidation reaction, commonly used to describe polymer degradation,^21,41^ typically involves molecular oxygen. It occurs slowly at ambient or moderate temperatures^42^ (Scheme 1).

This reaction is initiated by forming alkyl radicals (R•) via an initiator, which can generate metal residues, heat, or photocatalytic effects. Once formed, these radicals react with oxygen (O_2_) to produce several other radicals, including peroxyl radicals (ROO•) and hydroperoxyl radicals (HOO•). The ROO• radicals can further abstract hydrogen atoms from another polymer chain, forming hydroperoxides (ROOH). These unstable hydroperoxides decompose into various products, including H_2_O, alkenes, alcohols, ketones, and carboxylic acids. All of the expected functional groups were found here. We investigated whether Ag_2_O generates free radicals using electron paramagnetic resonance (EPR) spectroscopy.

### 4.4 EPR Spectroscopy and Proposed Mechanism

To elucidate the reaction mechanism of pentadecane decomposition, EPR spectroscopy was used to detect and identify possible radical species that form during the reaction. Initially, a temperature of 70 °C was selected due to the observed CO_2_ production at this temperature. The spectra of samples containing pentadecane and Ag_2_O measured at room temperature and 70 °C did not show significant signals, indicating that the intermediate radical species, if present, were too short-lived to be observed on the EPR time scale. For this reason, a spin trap was used, namely 5,5-dimethyl-1-pyrroline-N-oxide (DMPO). DMPO, similarly to other spin traps, is diamagnetic and, therefore, EPR-silent in its pristine state but reacts very quickly with radical species by trapping them and thus indicate by becoming paramagnetic. In this way, even very short-lived radicals can be observed and identified through the characteristic hyperfine couplings. The spin trapping mechanism, leading to the formation of DMPO adducts (DMPO-OOH as an example) is illustrated in Section SI and Figure S10. Typically, a low relative concentration of spin trap is added, therefore it can be safely assumed that it does not significantly participate to the chemical reaction, except for trapping the short-lived radicals.

**Figure 11:**
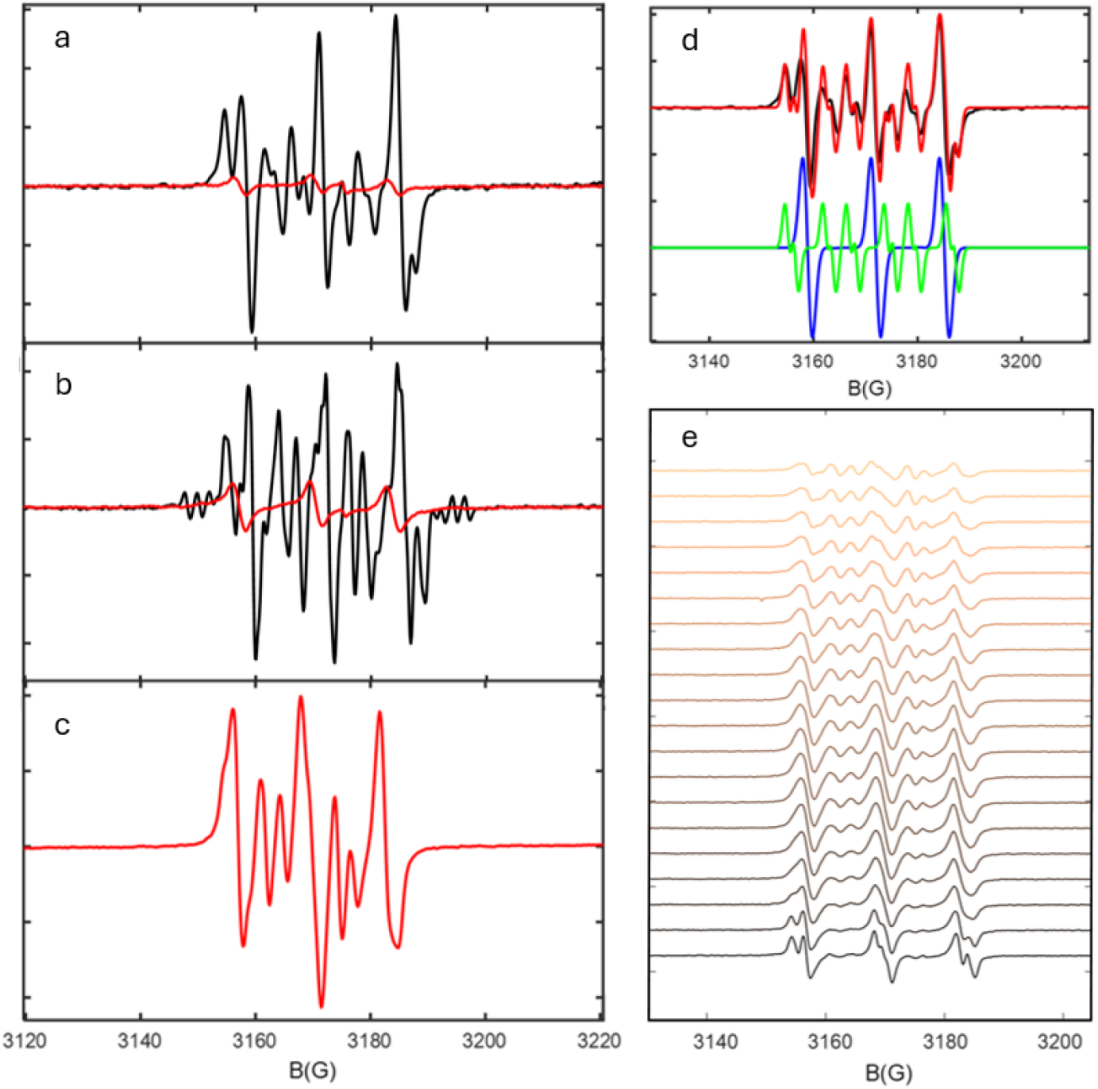
EPR spectra of DMPO-containing mixtures of pentadecane and Ag_2_O under N_2_ flow (black) and pentadecane opened to air (red) at different temperatures: room temperature (a), 70 °C (b) and 180 °C (c). d) experimental (black) and calculated (red) EPR spectra of DMPO-containing mixtures of pentadecane and Ag_2_O under N_2_ flow at room temperature, with single calculated components attributed to DMPOX (blue) and DMPO-OOH (green). e) Time-resolved spectra of DMPO-containing mixtures of pentadecane in air measured at 180 °C (time increasing from bottom to top), showing the formation of the DMPO-OOH/OOR adducts.

The EPR spectrum of a pentadecane/Ag_2_O suspension containing 10 mM DMPO under N_2_ gas flow showed a series of intense and partially overlapping signals (Figure 11a), indicating that a large number of radicals are formed during the reaction already at room temperature. Spectral simulations (Figure 11d) predict two signals characterized by different hyperfine couplings (Table 3). The first signal consists of a triplet characteristic for ^14^N hyperfine splitting, attributed to the oxidized DMPO, i.e., DMPOX.^43^ Since DMPOX typically forms in an oxygen-rich or strongly oxidizing environment, (Section SI and Figure S10) this further demonstrated the local oxidizing environment at the short-lived radical sites. The second signal exhibited one large and one small ^1^H coupling and the ^14^N coupling, which were diagnostic of trapped HOO• radicals. By comparison, pentadecane which reacted with the oxygen from air showed only a small residual DMPOX signal.

**Table 3:** Main DMPO Radical Adducts Identified by EPR Spectroscopy, with the Relative Hyperfine Constants (in MHz)

	DMPO-OOH	DMPO-OOR	DMPO-H	DMPOX
^14^N	34	34	40	38
^1^H	21	21	53	-
^1^H(OOH)	4	-	-	-

At 70 °C (Figure 11b) the spectrum of the Ag_2_O-containing sample was similar to the one measured at room temperature, but with a higher relative HOO• adduct contribution and additional weak signals at both higher and lower fields. Since the latter signals strongly overlapped with other components, its attribution was ambiguous. However, small ^14^N couplings were determined, suggested to be due to the DMPO-PNO adduct, the open-ring DMPO radical trapped by DMPO itself.^44^ This adduct commonly forms by further oxidation of DMPOX and is expected to form in even higher oxygen activity. Moreover, the features attributed to DMPO-OOH did not show a clearly resolved splitting from the smallest ^1^H hyperfine coupling. This is most likely due to the larger contribution of the DMPO-OOR adduct, which typically exhibits similar ^14^N and large ^1^H hyperfine couplings but lacks the weakly coupled proton. The pentadecane in the air sample showed a weak DMPOX signal at 70 °C. Only starting at 180 °C (Figure 11c, time evolution in Figure 11e) a DMPO-OOH/OOR adduct was present and a strong DMPO signal, similar to the presence of Ag_2_O already at room temperature. The spectrum of pentadecane-containing Ag_2_O could not be measured at 180 °C because of the fast Ag(I) reduction to Ag(0), leading to a strong increase in conductivity, which made the EPR measurement impossible because of the interaction between the electric field of the microwaves and the electric dipole of the sample. However, this observation confirmed that fast Ag_2_O reduction occurred at 180 °C, but the process is already active even at room temperature and becomes more pronounced at 70 °C. The results revealed that a free radical mechanism played the leading role in the reduction path and showed the existence of ROO• and HOO• species.

Generally, hydrocarbon oxidation in air at high temperatures (220–250 °C for pentadecane) occurs through free radical mechanisms,^21^ as observed in our reaction of pentadecane with air. Additionally, the reaction of Ag_2_O with pentadecane showed similar behavior already at room temperature.

One may recall that the near total of observed products of pentadecane decomposition were CO_2_ and H_2_O, indicating complete oxidative degradation of pentadecane molecules and accounting quantitatively for the final fate of the oxygen stemming from Ag_2_O. The formation of CO_2_ might occur through excessive oxidation via radical pathways, similar to a combustion process.^42,45,46^ The observed CO_2_ formation in the Ag_2_O-pentadecane reaction could therefore be facilitated by Ag_2_O acting simultaneously as a source of oxygen and a (auto) catalytic radical initiator.

### 4.5 Mechanism

The oxidation of hydrocarbons has been extensively studied across various fields, including polymer science,^47^ combustion chemistry,^19^ and biology.^42^ These oxidation processes typically proceed through radical pathways. However, the specific mechanisms can vary due to factors such as temperature, the presence of metals, and other reaction conditions that influence radical behavior. Complete oxidation results in the formation of CO_2_ and H_2_O at a characteristic ratio, while incomplete oxidation produces additional oxidized hydrocarbon products.^46^

In our studies with Ag_2_O-pentadecane, oxidation quasi-exclusively leads to the formation of CO_2_ and H_2_O as the main products, alongside small quantities of stable products such as alkenes, alcohols, ketones, and carboxylic acids.

Our main findings are thus:

1. The major byproducts are approximately 61% CO_2_ and 39% H_2_O.
2. The minor products of the reaction include alkenes and oxidized alkanes.
3. Combining pentadecane with Ag_2_O creates a synergistic effect that lowers both substances’ decomposition/reduction temperatures. As a result, the hydrocarbon decomposes into CO_2_ and H_2_O at a relatively low temperature, while silver oxide reduces into metallic Ag at temperatures near ambient (e.g., 70 °C).
4. While the thermal conversion of Ag_2_O to Ag in inert atmosphere or vacuum is typically an endothermic process, it becomes exothermic when mixed with pentadecane. At elevated temperatures, the reduction kinetics exhibits a sharp peak, further suggesting existence of a dominant reaction pathway.
5. Metallic Ag appeared in form of fused protrusions on the surface of Ag_2_O, and not elsewhere. An example could be seen in the supplementary video 1.

**Scheme 2:**
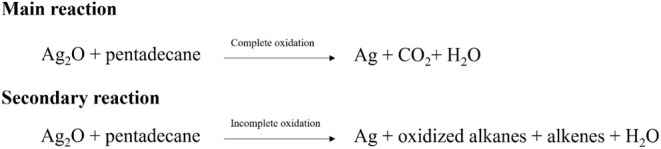
Schematic Summary of the Proposed Main Paths of the Reaction

One can thus summarize the main findings as follows:

A summary of the proposed majority and minority reactions is shown in Scheme 2. Initially, the presence of Ag_2_O promotes the formation of radical species at a low rate, as metal ions can generate free radicals.^22,38^ Furthermore, we observed two distinct types of byproducts: major (CO_2_ and H_2_O) and minor (oxidized alkanes and alkenes), suggesting two separate reaction pathways exist. The first resembles a complete combustion process where alkanes are fully oxidized to CO_2_ and H_2_O. Combustion reactions are exothermic, thus explaining why the response between Ag_2_O and pentadecane changes from endothermic with Ag_2_O alone to exothermic in the presence of pentadecane. This complete combustion suggests a highly oxidative environment related to the oxygen locally released from Ag_2_O. This oxygen may exist as highly reactive atomic oxygen atoms (O, highly reactive) or molecular oxygen (O_2_, a diradical species but less reactive than O). Atomic oxygen could be responsible for the low-temperature oxidation (CO_2_ release) observed at 70 °C. Additionally, the significant formation of DMPOX (detected during Ag_2_O-pentadecane reactions) further supports a highly oxidative environment, unlike the scenario when pentadecane reacts with O_2_.

Another possible explanation for the complete oxidation observed in the Ag_2_O-pentadecane reaction is the catalytic effect of Ag metal formed during the decomposition of Ag_2_O. Metallic silver (Ag) is known to catalyze oxidation reactions by facilitating electron transfer and enhancing the breakdown of hydrocarbon bonds.^38^ As Ag is formed in the reaction, this could reduce the activation energy of the oxidation process, accelerating the conversion of hydrocarbons to CO_2_ and H_2_O.

The second phase of the reaction involves partial oxidation, where alkanes are converted into alkenes, alcohols, ketones, aldehydes, and acids. Our findings suggest that in this phase, the oxygen from Ag_2_O behaves similarly to molecular oxygen, as evidenced by the detection of HOO• and ROO• radicals via EPR, along with oxidized alkane products identified through GC-MS, FTIR spectroscopy, and ^1^H NMR spectroscopy. These products are consistent with those typically seen in polymer oxidation, as outlined in Scheme 1.

Moreover, the nucleation and formation of metallic Ag appear to occur exclusively on the surface of the Ag_2_O, suggesting very limited mobility of silver in the alkane environment, rather than a solid–solid state reaction.

The proposed mechanism underscores the complexity of radical reactions in the presence of Ag_2_O. While most of the reaction leads to complete oxidation, a smaller portion results in partial oxidation. The dual role of Ag_2_O, acting both as a radical initiator (via Ag(I)) and as a source of reactive oxygen, introduces additional layers of complexity, which includes subtle effects due to particle morphology. Furthermore, the oxidation of hydrocarbons in the presence of Ag_2_O appears to be predominantly radical in nature.

## 5 Conclusion

In conclusion, we have demonstrated that Ag_2_O undergoes a redox reaction during its interaction with polyethylene. We established pentadecane as a model system that is transferable to the case of Ag_2_O in polyethylene melts around 200 °C. The primary byproducts of this reaction are H_2_O and CO_2_, with alkenes and oxidized alkanes appearing as minor products. EPR analyses disclosed the presence of radicals at 70 °C and even room temperature. We have also proposed a radical mechanism resembling complete combustion that agrees with the well-known hydrocarbon oxidation pathway.

The morphology of Ag reduced in hydrocarbons is dominated by a surface-confined solid–solid reaction. Several studies have highlighted the utility of this morphology in applications such as conductive welding. Since the radical mechanism controls this reaction, a radical initiation/quenching could readily adjust the transition temperature. Besides applications in reactive extrusion or electric welding, these insights contribute to a broader fundamental understanding of the reaction of Ag_2_O with hydrocarbons.

## Supporting Information Available

A video demonstrates how silver oxide can reduce into silver under ethane gas, through a solid–solid surface reaction. Conversion of silver oxide to silver was captured in a Quattro S SEM (Thermo Fisher Scientific, USA) Environmental Scanning Electron Microscope (ESEM). A home-built heating stage is used, with silver oxide pressed into an aluminum crucible. The crucible’s base is laser-heated, with temperature monitored by a thermocouple. Under an ethane atmosphere of 34 Pa, the sample is heated from room temperature to 300 °C at 6 °C/min (AVI)
Thermal characterization of silver oxide and silver oxide with pentadecane, reaction setups, GC results of H_2_O production, prove that pentadecane does not produce CO_2_ without silver oxide, GC-MS result of the liquid adduct of the reaction of silver oxide with pentadecane, mass spectra of 1-hexene, and formation mechanisms of the oxidized DMPO species (PDF)

## Supplementary Material

am5c01780_si_001.avi
