# Supplementary material for: Silver Oxide Reduction Chemistry in an Alkane Environment
Source: ACS Appl Mater Interfaces. 2025 Apr 29;17(19):28808–21. doi: 10.1021/acsami.5c01780 (PMC12086840; doi:10.1021/acsami.5c01780)
Supplement: Supplementary file 2 — am5c01780_si_002.pdf [file am5c01780_si_002.pdf]

Supporting information

Silver Oxide Reduction Chemistry in an Alkane Environment

AUTHOR NAMES

Fayez Alfayez[a,b], Mikhail Agrachev[c], Fabian Matter[b], Sandro Lehner[a], Arvindh Sekar[a], Walter Caseri[b], Rudolf Hufenus[a], Sabyasachi Gaan[a,], Manfred P. Heuberger[a,b,*]*

AUTHOR ADDRESS

[a]Department of Advanced Fibers, Empa Swiss Federal Laboratories for Materials Science and Technology, St Gallen CH-9014, Switzerland

[b]Department of Materials, ETH Zürich, Zürich CH-8093, Switzerland

[c]Department of Chemistry and Applied Biosciences, ETH Zurich, Zürich CH-8093, Switzerland

Emails; Sabyasachi Gaan sabyasachi.gaan@empa.ch; manfred.heuberger@empa.ch

SUPPORTING INFORMATION

Figure S1 details

The Ag_2O sample was analyzed using thermogravimetric analysis (TGA) under N_2, which indicated minor weight loss between 120 ˚C and 240 ˚C (Figure S1), likely due to decomposition of Ag_2CO_3 contamination. The onset of reduction was observed at 400 ˚C. A significant weight loss, approximately 6.95%, occurred between 350 ˚C and 410 ˚C, closely matching the theoretical value of 6.90% for the conversion of Ag_2O to metallic silver. This strong correlation confirms that the weight reduction corresponds to the decomposition of Ag_2O. Based on these results, the Ag_2O was oven-treated at 220 ˚C for 5 h.

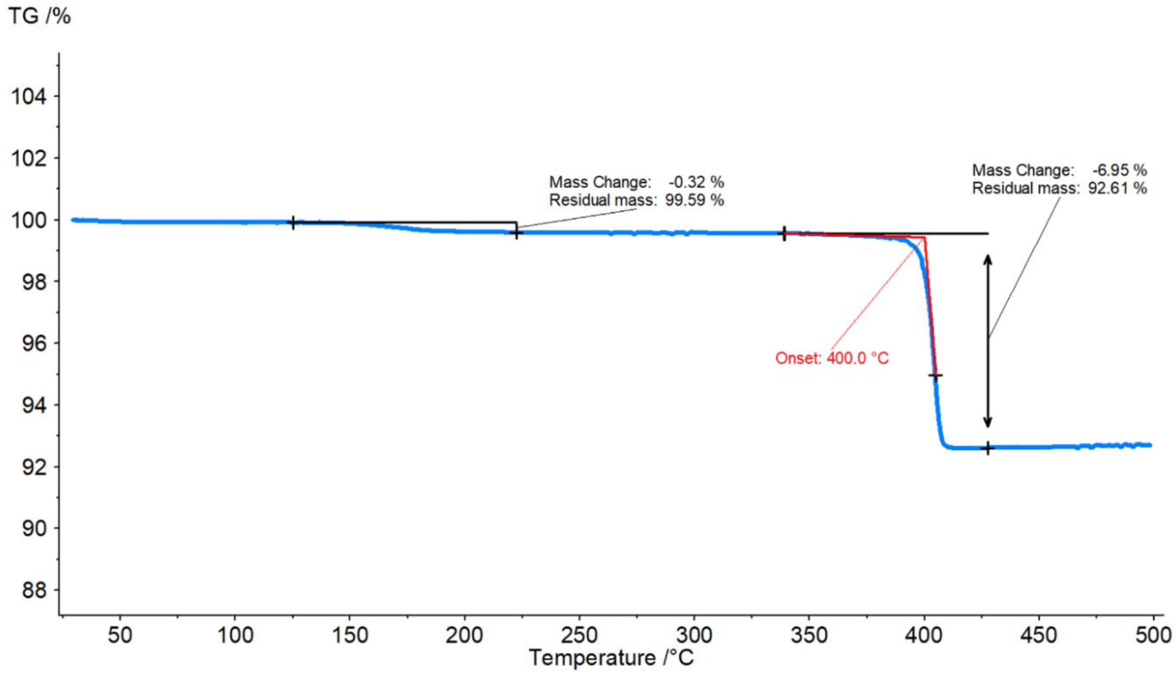

Figure S1. The TGA curve shows a gradual weight loss of pure Ag_2O as the temperature increases. Significant weight losses are observed at temperatures around 400 ˚C, leaving a residual mass of 92.61% (theoretical 93% for complete loss of oxygen). The heating rate is 10 ˚C/min and the test was run under 50 mL/min N_2 flow.

Figure S2 and S3 details

Samples of Ag_2O and pentadecane were heated at varying rates (Figure S2). Using the Ozawa-Flynn-Wall method, the activation energy was calculated to be 81.85 kJ/mol. Additionally, this method was employed to predict the conversion yields versus time at specific temperatures (Figure S3), providing the reaction kinetics.

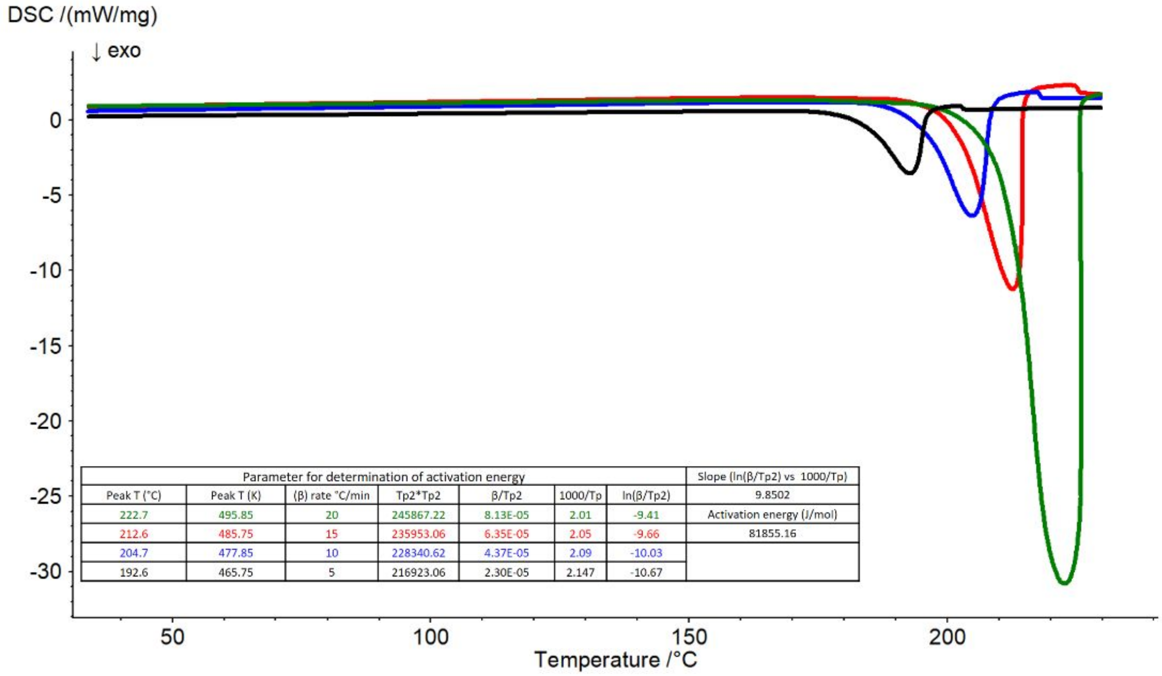

Figure S2. 5 mg of a 1:1 mole ratio of Ag_2O and pentadecane sample was analyzed at different heating rates of 5, 10, 15, and 20 ˚C/min (from 25 to 230 ˚C) under a N_2 flow of 50 mL/min. The table presents the parameters used for calculation of the activation energy.

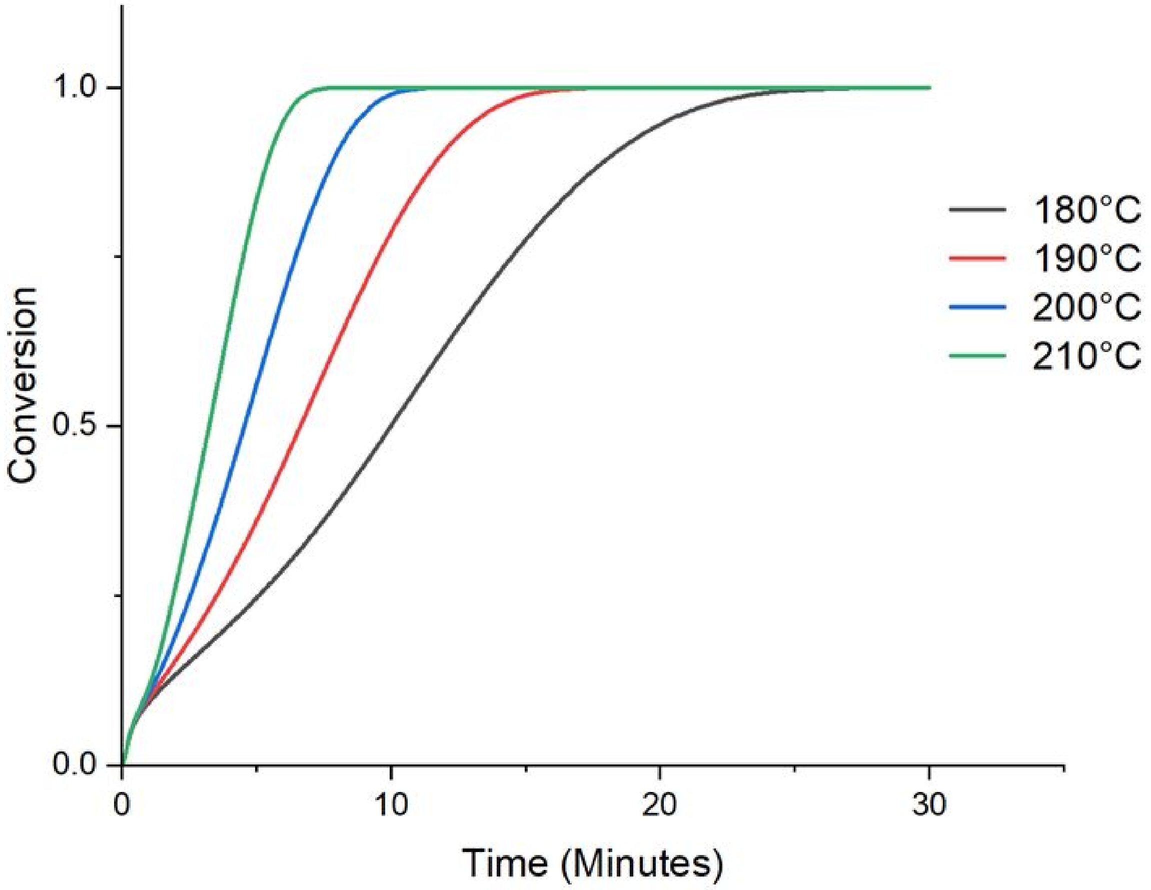

Figure S3. Predicted reaction time using the Ozawa-Flynn-Wall method.

S4 and S5 details

To capture the byproducts, the setup shown in Figure S4 was adopted. Furthermore, the setup used to quantify the amount of CO_2 is depicted in Figure S5. In this, an argon carrier flow (20 mL/min) was bubbled through the stirred reaction solution at a constant temperature of 145 ˚C, which is deliberately selected below the peak transition temperature (i.e., 193˚C) to obtain a slower conversion and allowed for the acquisition of more data points for integration. The gas outlet was connected to a GC (Micro GC Fusion, Inficon, Switzerland) equipped with three columns: Rt-Molsieve, Rt-QBond, and Rt-U-Bond (Restek, USA). The gases then flowed at a set rate of 20

mL/min to the TCD detector, with each measurement interval taking 2 min. The TCD detector was calibrated to detect the gases of interest, and the amount of CO_2 produced was quantified by integrating the area under the curve of concentration versus time.

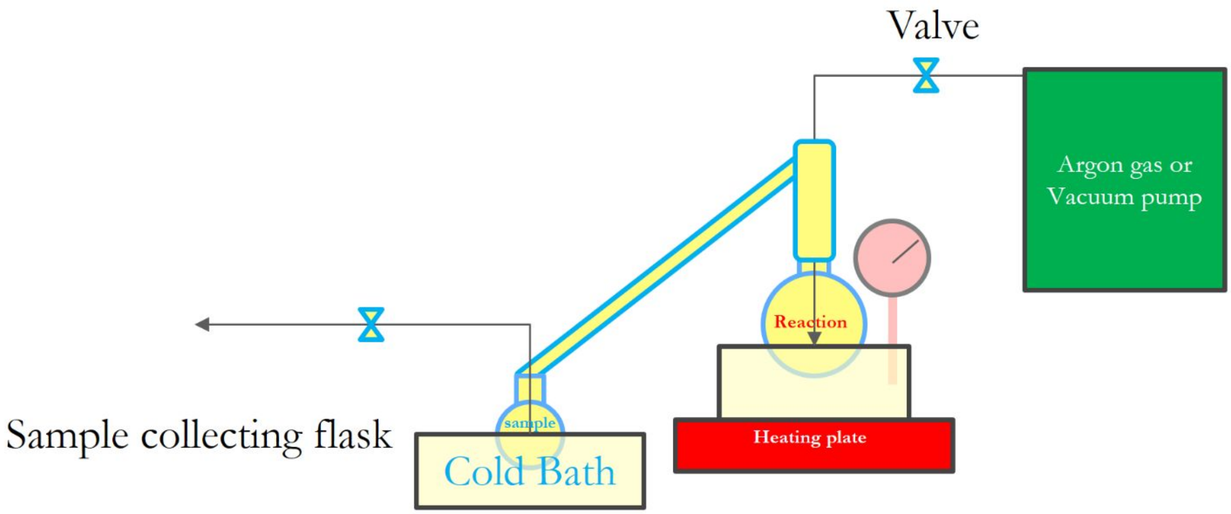

Figure S4. The setup used to collect reaction vapors. The reaction flask was kept at 180 °C for 30 min. the cold bath (acetone/ice slurry) was maintained at −78 °C. a constant flow of Ar.

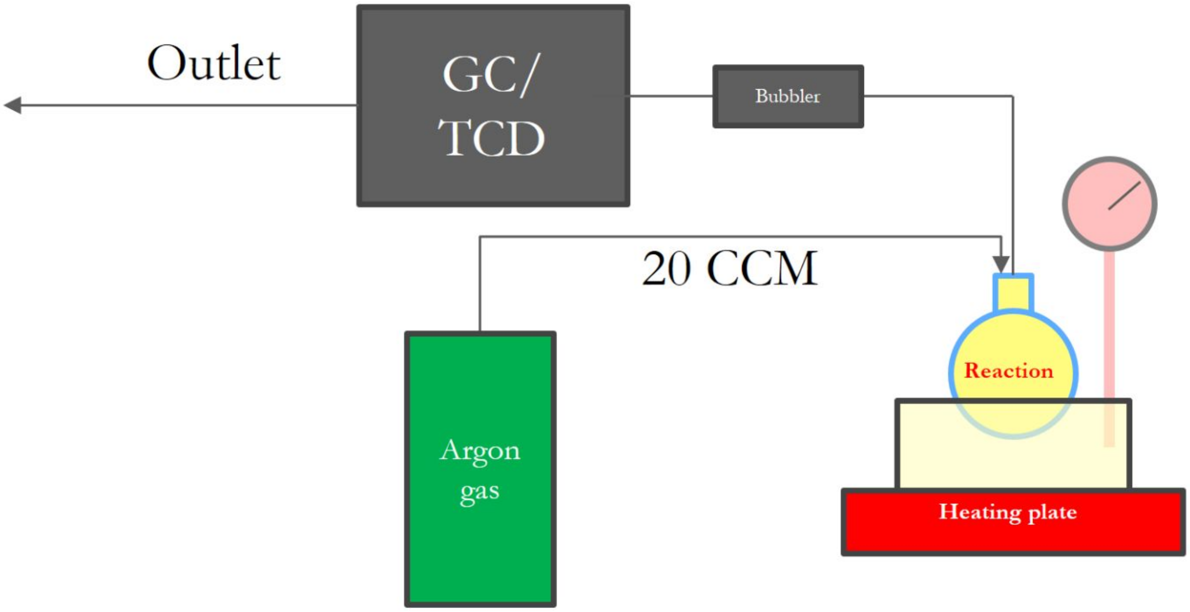

Figure S5. Schematic representation of the GC/TCD setup used for gas measurements, purge flow is set at 20 mL/min.

S6 details

From the GC-TCD measurements, an H_2O signal was observed (Figure S6). However, the setup was not designed to quantify the amount of H_2O.

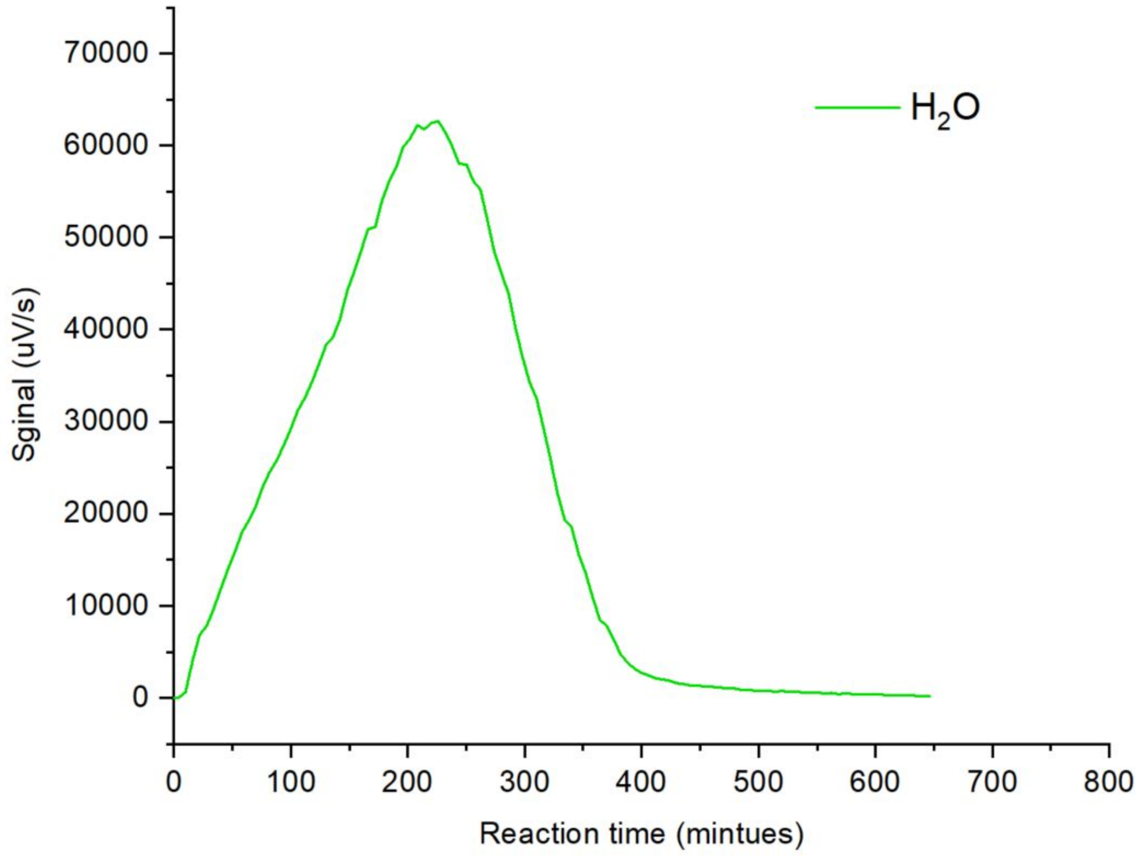

Figure S6. H_2O signal intensity versus time, observed in the reduction of Ag_2O in pentadecane at 145˚C using GC-TCD (sampling rate: 2 min). Although the system was not calibrated for H_2O, signal strength directly relates to the concentration of H_2O in the gas stream.

S7 details

A 0.2 mL sample of pentadecane was placed in a 20 mL vial, prepared in open air. The vial was then sealed and placed in the GC-MS agitator. The agitator heated and agitated the vial at 70 ˚C for 120 min. After this period, a headspace sample was taken to measure the amount of CO_2 produced. The amount of CO_2 present in the air was also measured as a control. The results showed that no CO_2 was produced when pentadecane was heated in the presence of oxygen.

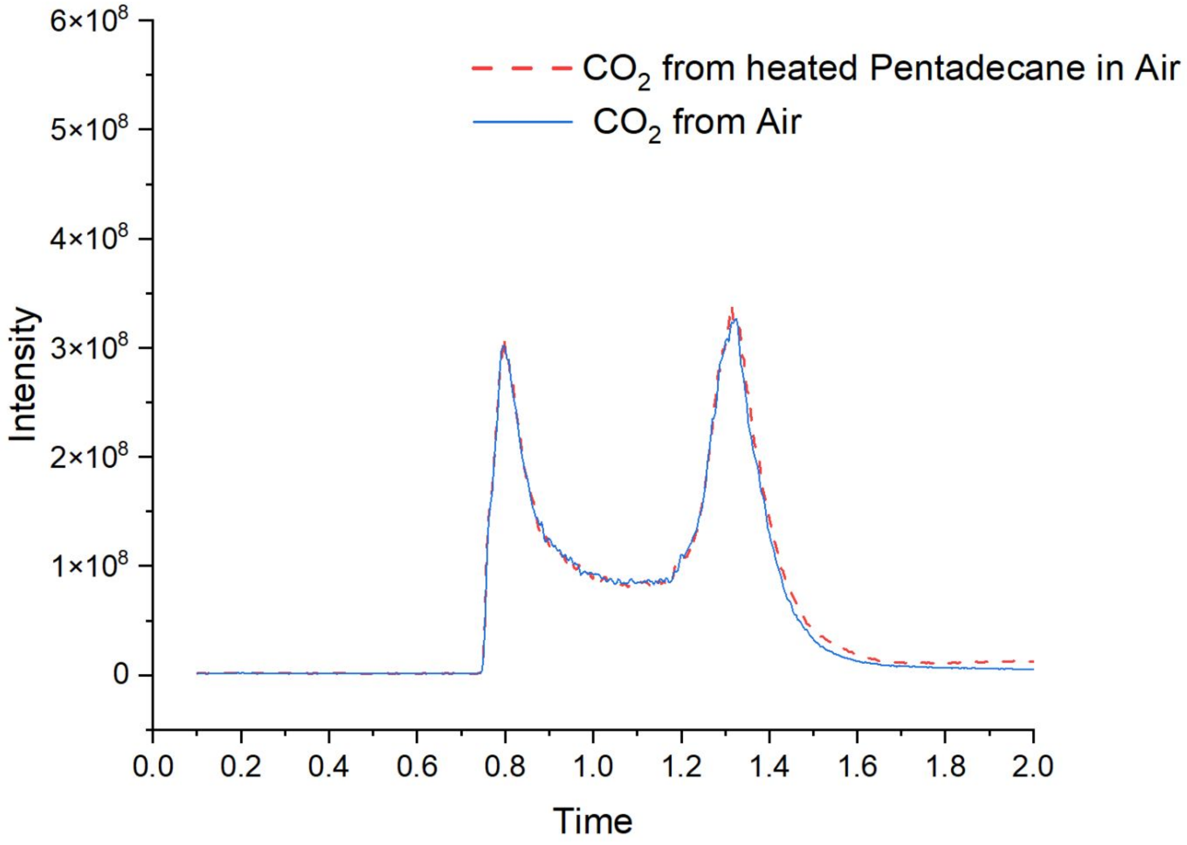

Figure S7. GC-MS measurements of two samples, highlighting the region associated with CO_2 (44 m/z). Pentadecane was heated at 70 ˚C in a vial containing air for 120 min (red dotted curve). Meanwhile, a vial containing only air was measured to display the background CO_2 present in the air (blue curve).

S8 details

The adduct formed by the reaction of Ag_2O with pentadecane was analyzed using GC-MS. The analysis revealed no detectable changes in the pentadecane (Figure S8).

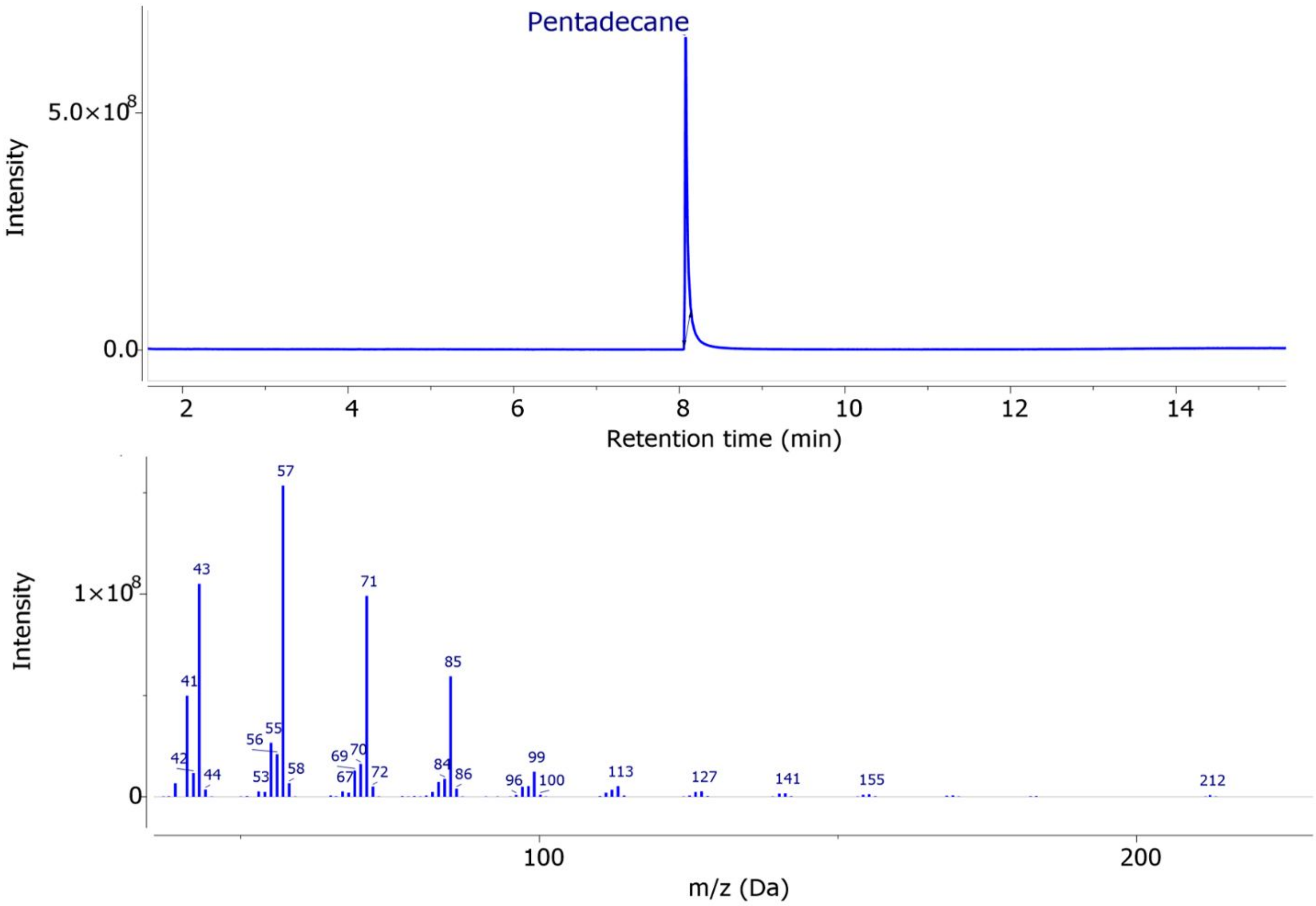

Figure S8. GC-MS analysis of the liquid adduct from the reaction between Ag_2O and pentadecane showed the presence of pentadecane only, with a retention time of 8 min and a molecular ion peak at 212 m/z.

S9 details

The mass spectra of a peak with 1.755 min retention time, from the experiment shown in Figure 10 as an example of a 1-alkene, specifically 1-hexene (Figure S9).

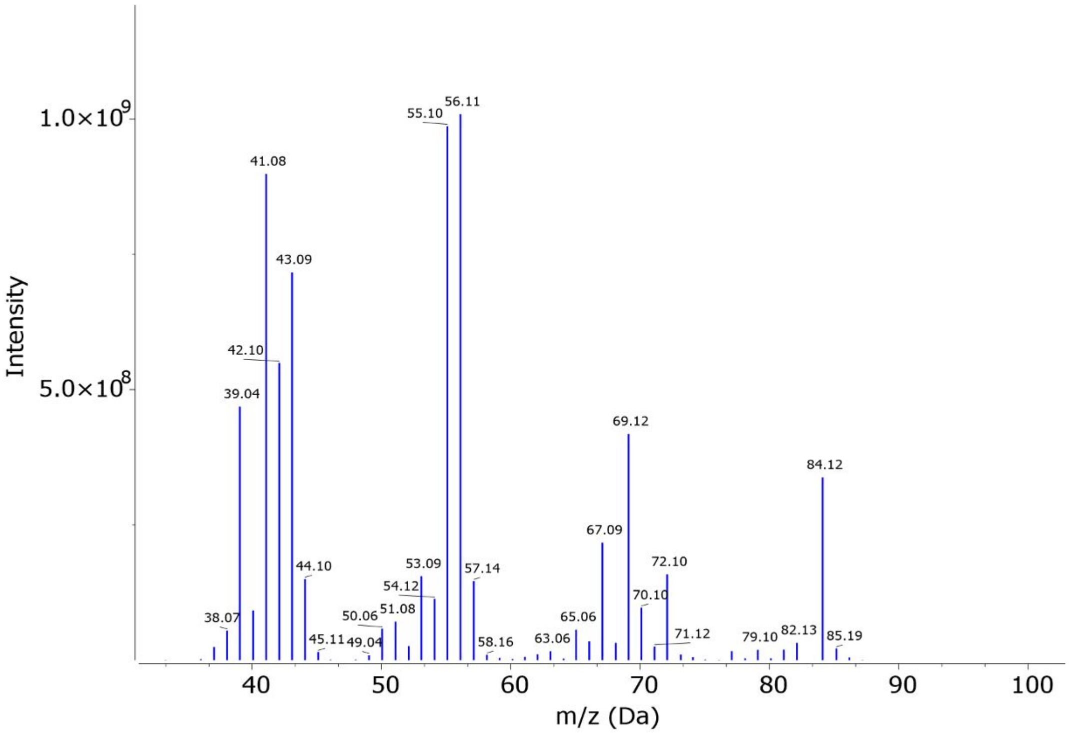

Figure S9. Exemplary mass spectrum of 1-hexene with a retention time of 1.755 min. The main peaks are observed at 84, 69, 56, and 41 m/z.

S10 details

Illustration of the formation of the DMPO adducts (DMPO-OOH, DMPOX and DMPO-PNO) is shown in S10.

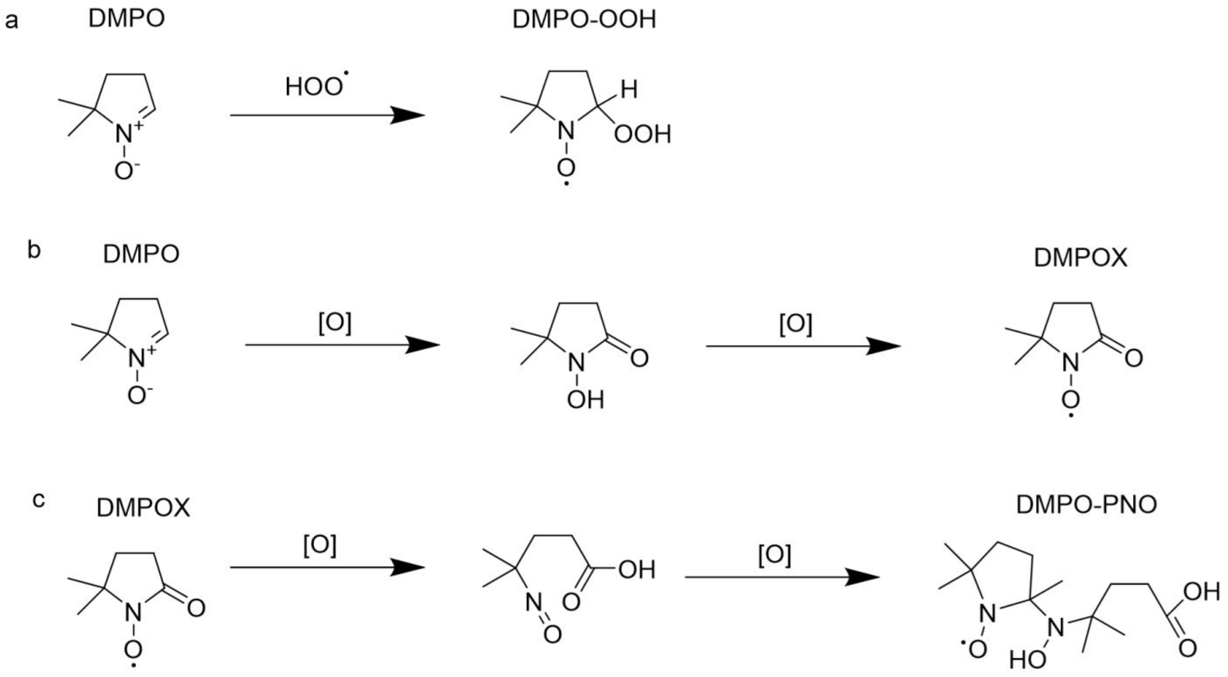

Figure S10. Formation mechanisms of (a) DMPO-OOH (analogous for other adducts, such as DMPO-OOR, DMPO-OH, DMPO-H etc.), (b) DMPOX and (c) DMPO-PNO.
